# Spirit of the British and American Periodicals, &c.

**Published:** 1844-10-01

**Authors:** 


### Spirit of the British and American Periodicals.

STATISTICS AND CASES OF MIDWIFERY; COMPILED FROM THE RECORDS OF THE PHILADELPHIA HOSPITAL, BLOCKLEY. By GEO. N. BURWELL, M.D. (From the American Journal of the Medical Sciences, April, 1844).

The cases of labour contained in this article occurred from October, 1835, to January, 1844 inclusive, a period of eight and a half years.

The duration of pregnancy given in these tables corresponds very well with that ordinarily given. The relation between the time of the last menstruation and the birth of the child is an interesting one. It was noted in 259 cases, as follows:—

| | | |
|---|---|---|
| In 1 case | menstruation occurred about | 5½ months before delivery. |
| 4 cases | . . . . . | 6 |
| 1 case | . . . . . | 6½ |
| 9 cases | . . . . . | 7 |
| 2 cases | . . . . . | 7½ |
| 24 cases | . . . . . | 8 |
| 24 cases | . . . . . | 8½ |
| 138 cases | . . . . . | 9 |
| 26 cases | . . . . . | 9½ |
| 18 cases | . . . . . | 10 |
| 11 cases | . . . . . | 11 months and over. |
| 1 case | . . . | at irregular intervals throughout gestation, |

the appearance of the catamenia being preceded by violent neuralgic pains.

The points of interest in this table are 1st, the comparatively small number of cases where the intervening time has been less than 8 months; 18 in 259 cases, or in the proportion of 1 to 15; of these 18 cases, labour came on in 9 of them at 7 months. This large proportion taken in connexion with the results would seem to indicate a disposition of Nature to expel the child at the seventh month of utero-gestation. The question would arise also whether the presentation of the child had any thing to do with this. The case at 5½ months was a woman who dated her pregnancy nine months before delivery, but who continued to menstruate regularly until this short period before accouchement—child healthy and weighed 6 pounds. In the cases at 6 months the children were all born alive, and had the average weight of nearly 6½ pounds. The second point of interest in this table is the large number of cases at eight and eight and half months. The natural duration of gestation being 9 months, it must follow that these were either premature or the catamenia must have appeared once after conception. The number of cases of labour where menstruation had not taken place for eleven months or longer previous to delivery, is sufficient to shew that regularity of the catamenia is not always necessary to conception. There is one case in this number where the patient had not menstruated for eleven years previously.

*Twin Pregnancies.*—Of 588 labours there were 598 children.

*Sex.*—This was noted in 517 cases as follows: males 277; females, 240.

This gives a percentage of 53 per cent. males, and 47 per cent. females.
Dr. Churchill's tables give 59 . . . . . . . . . 41

Registrar General's of England Report gives 51 per cent. males and 49 per cent. females.

*Weight.*—The average weight of 100 male children at full term was 7lb. ¾ oz. Of 100 female children it was 6 lbs. 8½ oz.

*Still-born Children.*—Of the 598 children the large proportion of 54 were dead at birth, or lived only a few hours afterwards.

With respect to retention of the placenta the dates were not sufficiently definite to render any inference regarding it positive.

*Hæmorrhage.*—Thirty cases of hæmorrhage—twice, before the delivery of the child—15 times before the delivery of the placenta, and after the birth of the child; and 13 times after the expulsion of the placenta.

*Convulsions.*—13 cases during progress of the labour; of these 4 were epileptic; 4 apoplectic; 1 a complication of hysteria with epilepsy; 1 partly cataleptic and partly epileptic.

---

### MENINGITIS—HOMŒOPATHY.

A case of this kind was related by Mr. Adams lately before the Dublin Pathological Society. A young lady, aged 23 years, complained on Christmas-day of sickness and chilliness. Two days afterwards she complained of double vision. On the 28th, she was visited by an eminent Professor of Homœopathy, who undertook the case, as one of fever, which was going on well. Next day she had a convulsion, followed by delirium, &c. We need not notice the symptoms or the treatment. The former were unequivocal of dangerous inflammation in the brain—the latter worse than no treatment at all. The patient craved for fruit, whey, chicken-broth, &c., but these were not allowed, and the bowels were never opened till the day before she died. On dissection, the membranes were highly congested, and the pia mater quite red. Much bloody serum was found in the ventricles and in the spinal canal. The base of the brain was like scarlet cloth!

It has been said that the only charge against homœopathy is the loss of precious time in acute diseases. But we are justified in charging homœopathy with gross ignorance of pathology or the nature of disease. What man capable of interpreting the symptoms of a malady, could think of treating meningitis by tea-spoonfuls of water, without any efficient medicine of any description? The truth is, that the homœopathists are wholly incapable of recognizing acute diseases; and they therefore treat them all as chronic, or rather as imaginary affections—that is, with low diet and no medicine!

---

### PROTRACTED LACTATION.

With the exception of a case by Dr. Kennedy in this Journal, the following is one of the most remarkable we have met with of Galactirrhœa. It is related by Dr. Green in the September number of the New York Journal.

Mrs. T. V., aged 47 years, and the mother of four children, has had abundance of milk for 27 years past. There has been a period of exactly four years and a half between each birth, and the children were allowed to take the breast till they were running about at play. She had now been nine years a widow, and is obliged to have her breasts drawn daily, the secretion of milk is so copious. The distance between the periods of pregnancy is worth the consideration of our political economists, and illustrates the doctrine of Dr. Loudon on the subject of protracted lactation.

---

### ON THE ISOLATED TUMOR (OR TUBERCLE) OF THE UTERUS. By Dr. ROBERTS.

The fleshy tumor of the uterus is, according to our author, more frequent than is generally supposed, and is often the cause of complaints that go under other names, as prolapsus uteri, menorrhagia, hysteralgia, leucorrhœa, dysmenorrhœa, &c. These are often effects of the tumor, which is more frequently seated in the posterior walls of the organ than in any other part. The following cases are illustrative of the subject.

"*Case* 1.—Mrs. Easter, widow, aged 43, mother of six children. Began to have pain in the womb within the last two years. Her principal complaint of pain is in the abdomen, low down in the groin, crossing the belly and settling in the hypogastric region, attended with bearing down and pressing, and a feeling that if something would come away she would be better. The pain is not constant. Sometimes it comes on in short and sudden paroxysms of great severity; sometimes lasts for hours, and sometimes for two or three days. Lying down relieves it; it is increased by lifting or straining, particularly at stool. Sometimes she is afraid to walk, owing to the bearing down in the lower part of the belly. Sitting down at all times causes pain in the left side. She has sundry dyspeptic symptoms. On examination, *per vag.*, a tumor as big as a hen's egg is felt to rise from or in the posterior wall of the cervix uteri, about half an inch above the lip of the os, having around it a distinct sulcus, by which it is circumscribed. It extends towards the left side of the patient; is elastic, rounded, and has a roughened feel on its surface, as if formed of flattened convolutions, divided by shallow sulci, but is not distinctly nodular. It is exquisitely tender, and the examination left a pain (which she describes as beating, hot, burning, dull, or numb) evidently neuralgic, to which much of the distress is doubtless owing, which lasted some time.* In all other respects the uterus appears healthy; it is rather low, and the neck is tilted forwards towards the pubis. Such was the history of the case some months ago. The symptoms still continue, but I have made no recent examination, nor has any treatment been submitted to.

"*Case* 2.—Mrs. Derest, aged 24. Seen July 28, 1837. Enjoyed good health previous to her marriage, four years ago, and never since. Had two miscarriages: no living children. Has been suffering much for some months. Pulse one hundred and tense; bowels costive, urine stringy and passed with pain. The inguinal and hypogastric regions are very painful on pressure, but no tumor can be felt from without. There is a good deal of pain in the back, a sensation of bearing down, and of something obstructing the rectum and the evacuation of fæces, and occasional paroxysms of dreadful pain, lasting for hours. On examination, the uterus was found prolapsed, its os and cervix quite healthy. On passing the finger behind the cervix, and upwards towards the left side (that which is the most painful), a hard, knobbed enlargement is to be perceived; and, *per anum*, a large tumor can be felt, occupying the hollow of the sacrum, and pressing towards each other the walls of the rectum. The body of the uterus seems enlarged. The examination was followed by severe pain, which lasted several hours. The woman was a patient of Dr. Jas. B. Kissam's. Of the subsequent events of the case, I know nothing, except that she took iodine, and I heard lately that she was still living, and in better health. Since the above was written, I found the following memoranda relating to the patient among my papers. Nov. 26, 1840.—After taking 3 oz. Sol. Iod., considered herself well for a period of two and a half years. The symptoms (the same as before) are again as bad as ever. The extreme tenderness of the posterior lip of the uterus renders the examination of the tumor difficult, but it is certainly smaller than formerly. The anterior lower portion of the body of the uterus is enlarged, irregular, in parts harder than in others, and exquisitely tender to the touch; towards its left side it is pretty distinctly circumscribed as a tumor, but accuracy

* "The connection between hysteralgia and fibrous tumors of the uterus, has been ably pointed out by Ingleby (Lect., p. 204). He has known of four cases thus caused. In a case of small hard tumor of the upper part of the cervix, extreme tenderness of the vagina was a marked symptom."

of examination is forbidden by the pain which is caused by the touch. Jan. 1, 1841.—Much relief from the use of Iod. Hydrarg. August, 1844.—No examination since.—Health perfectly good.

"*Case* 3.—H. Green, light mulatto, aged 28 years: seen 9th July, 1837, Married 12 years: two children by her first husband, none by her second—none for several years past. Is stout and active, menstruates regularly but scantily. urinates freely as to quantity, but has occasionally extreme pain at the end of the urethra, after the evacution, causing her to shed tears even. Has also pain in the loins. The belly is not tender, and no tumor can be felt in it. The cervix uteri is hard and stiff, and is, as it were, tilted up towards the opposite side from the tumor. The outer surfaces of the lips of the os are rough, hard, and irregular; and the os itself has the shape of an *S*. (The speculum was not used.) The finger, *in ano*, encounters a large tumor springing from the posterior surface of the uterus, just above the cervix, which is hard and irregular, but does not block up the rectum as much as in the preceding case. There are some circumstances connected with this case which are highly interesting, in the history of the disease. The tumour *disappeared* very rapidly under the use of iodine, not a vestige of it being left, and for some years I considered the cure as complete. About a year ago I obtained permission to examine the uterus, and was much pained to find that a tumor of some size occupied the site of the former one; in other words, *that it was growing again.* As no complaint was made at the time, I said nothing about it; but on the 12th of March last, was again consulted, for a return of the old symptoms. A very considerable tumor now occupies the posterior surface of the uterus, reaching towards the sacrum, having a flattened feel, its lower edge rather sharp, tolerably smooth, and rather painful. The function of the uterus is not impaired. Further to increase the probability that these tumors belong to the fibrous variety, a very distinct one is to be felt in the right groin, tender, nearly as large as an egg, and apparently attached to the organ. This latter tumor is much less painful to the touch since leeches were applied over it. *The disappearance and subsequent return of the disease* are further exemplified in case 4th.

"*Case* 4.—Mary Stevens, colored, aged about 30, married 12 years, and mother of two children, consulted me for pain in utero, prolapsus, and scantiness of the catamenia, Sept. 1837. The lower portion of the posterior wall of the uterus was then swollen and tender. In October, there existed a tumor of considerable size, which, on the 28th November, was recognized by my friend, Dr. G. W. Hodgson, now of White Plains. *Per rectum*, it could be felt to consist of two rounded portions, as large as walnuts, divided by a central sulcus, and very tender. Under the use of iodine, they became smaller, softer, and more circumscribed, and could be pushed up with the uterus into the abdomen. She complained of pain, weight, and leucorrhœa. On the 1st of May, 1838, the tumor was as large as a walnut, softer, and smooth. During the next six months, it rapidly increased in size, being nearly as large as a small orange. On the 28th March, 1839, she called, stating herself to be some months advanced in pregnancy, and, to my surprise, *I could not discover the tumor.** She was delivered easily, in due time, of a living child. Some months afterwards, on examining her again, I felt the tumor, elastic, as large as a walnut, of circular shape, very tender on pressure, and occupying its original site. There was a great degree of prolapsus; about this time she was examined by Doctor (now Professor) Gilman. The tumor increased, and is now of considerable size, and causing a good deal of uneasiness, but of late has not appeared to enlarge. A year ago, when a patient of the Northern Dispensary, for dysmenorrhœa, which appeared

* "A case of spontaneous disappearance of two large fibrous tumors is to be found in the first vol. of 'Clark on the Diseases of Females,' 1814, 1st ed., p. 250.

to subside under the use of the bougie, she was several times examined by my able and zealous colleague, Dr. James Crane, Jun.

"The symptoms which *may* arise from the disease now under consideration, are further illustrated by the following :—

*Case* 5.—"Mrs. Sands, mulatto, aged 20, small and delicate; had been married six weeks. On 19th December, 1839, was exposed to cold: on the 20th, menstruated, as she had always done, with pain, which continued severe until the evening of the 23d, when it abated under the use of anodynes. On the 25th the pain recurred, and she vomited up everything she took. On the next day, general peritonitis existed in a well-marked form, and yielded to the usual treatment. The digestive organs continued to be disordered for some time afterwards, and occasional attacks of pain and tenesmus occurred. On the 13th January she began to complain of a return of the old pain, recurring at frequent intervals, at times distressingly severe. On these occasions she walked about her room, bent double, and almost delirious with agony. I now made a vaginal examination. The uterus was very much prolapsed, the neck tilted forwards, thick and firm, and the os gaping. A large round tumor, springing from the posterior wall just above the cervix, as large as a small orange, of uniform smoothness, hard, and very painful on pressure, occupies the vagina and encroaches considerably upon the rectum. The examination caused pain. The pubic region was tender. She had no particular difficulty in urinating, or in defecation, but had long been annoyed by an inclination to expel something from the rectum; otherwise, previous to her marriage, she had had good health. Every evening, from the date of the examination, until the 28th, she was attacked, about 5, P. M., with excruciating pain in the abdomen, beginning in the groins, during which she tossed about in bed in all directions, imploring the administration of her anodyne (McMunn's El. Op.), which invariably speedily relieved. The tumor soon disappeared under the use of iodyne, but what has been her condition since I have had no opportunity of determining.

*Case* 6.—"Mrs. Rankins, *æt.* 30, married 5 years: mother of two children, visited Mar. 4, 1840. Her husband had been absent for thirteen months; immediately on his return, she was attacked with excessive pain in the uterus (having previously enjoyed good health), followed by symptoms of peritoneal inflammation, requiring depletion and anodynes. On the subsidence of the more serious symptoms, she lay in a languid, suffering and highly nervous state until the 1st July, when on a vaginal examination the uterus was found prolapsed, and a tumor was detected, circumscribed and as large as a lady apple, on the posterior wall of the uterus, and toward its right right side. It had the usual elastic feel, and was very painful on pressure, appeared as if lobulated externally, and was very distinct per rectum."

*Case* 7.—"Mrs. Johnson, aged 36, mother of one child, applied to me about one year ago, for certain uneasy sensations about the back, hypogastrium, &c., which led to the discovery of an enlargement in the posterior part of the uterus, of some size, but the particulars of which I cannot recall to my memory. Subsequently she disclosed a large fluctuating tumor upon the right loin, which, on being opened, contained a thin purulent fluid derived from carious bone beneath. The fistula still discharges, and I regret to add that a similar tumor, as large as my two fists, now exists in the left groin and is rapidly enlarging. Notwithstanding, her general health is good. The tumor of the uterus, examined this day, Aug. 3, has lost much of its original circumscribed character, and feels more like a general enlargement of the posterior surface of the organ, increasing in thickness as it ascends, hard, not very irregular, and excessively tender, so as to render the examination difficult. It arises from about half an inch above the os, the portion of neck which is sound not being tender; the uterus is low, its anterior surface healthy. It does not interfere with defecation; menstruation is easy and regular, but scanty; leucorrhœa habitual, and coitus so painful as to be allowed only with the greatest reluctance. The examination, as well as it, lead to aching pain afterward, from hip to hip and through the body. There is pain in the back and belly, and the least jar in walking causes her to scream out. Some of this may be due, however, to the caries which exists in the pelvic bones. She has taken a good deal of iodine at irregular intervals, to which, perhaps, the change in the tumor may be owing. Several more cases have come under my observation in private practice; two or three such were seen by myself and Dr. Crane in the Northern Dispensary, and the latter also saw a case in private practice. Prof. Gilman has also met with and treated such an one, successfully. As I am not writing a history, but a sketch only of the affection, I shall leave the symptoms to be inferred from the cases which I have now related, remarking only that they present nothing pathognomonic, but are the common expression of several uterine affections. That each may exist separately and from other causes, I well know, but I leave it to the profession to decide, how far, when they coincide, they are to be considered as in the relation of cause and effect, and how far the removal of the tumor demands our special attention. The neuralgic character of the pain with which they are apt to be associated, merits a passing notice, not less than their frequency, the uncertainty of their nature, their tendency to remain stationary, to grow slowly, or rapidly, to disappear and spontaneously to return, *their peculiar predilection for the posterior wall of the uterus*, their innocuousness to life, and their insidious development, until revealed under the influences of some stimulus, of which matrimony and the renewal of coitus are conspicuous. Of the causes of the disease I know nothing; for the prognosis, I refer to the quotation from Lisfranc, and leave the diagnosis to the tact and judgment of the reader. A very few words respecting the treatment.

"The only remedy for these tumors on which any reliance can, in the present state of our knowledge, be placed, is iodine."

---

On the Extraction of the Retained Placenta in Abortion. By Henry Bond, M.D. (From the American Journal of the Medical Sciences, April).

All authors are agreed that, in cases of abortion, the hemorrhage is kept up by the presence of the placenta, and that, as soon as this is removed, the hemorrhage always ceases—they do not suppose that the placenta is retained by any firm adhesion, but for the want of some means to reach it with safety, and get a secure hold of it. When the placenta projects into the vagina, the introduction of one or two fingers will suffice to extract it. But in the most troublesome and dangerous cases, where the placenta is inclosed within the body of the uterus, and its neck is contracted, the finger can do nothing. Dr. Bond here enumerates a variety of instruments invented by different accoucheurs for the removal of the placenta in such cases, and after pointing out the objections to each, he suggests one of his own invention to which he gives the name of *placental forceps*. This instrument is about 10 inches long, curved laterally on a radius of about 12 inches, and the blades about 1½ inch longer than the handles. The blades terminate in an oval expansion nearly half an inch wide. The handles and blades, including the edges of the oval expansion, are rounded and bevelled off, so as to preclude all possibility of injuring the surrounding soft parts. The inner part of the oval expansion is made concave and rough, so as to maintain a severe grip on the body embraced. The outside of the oval part of the blades is made slightly concave and smooth, without a fenestra, so that, in passing them through the os uteri, and expanding them so as to embrace the placenta, there shall be the least danger of injuring the part.

### On Perityphlitis, or Inflammation of the Cellular Tissue adjacent to the Cæcum. By Wm. Seller, M.D. &c. (Abridged from the Northern Journal of Medicine, July 1844.)

This disease, which is characterised by inflammatory turgescence adjacent to the cæcum, has its seat external to the peritoneum, in textures of a cellular structure, and, unless neglected at first, is more prone to resolution than to the formation of pus; when suppuration does occur, the pus is almost invariably evacuated by the rectum. Tenderness on pressure, hardness, dulness on percussion, and circumscribed swelling of the abdomen, adjacent to the anterior part of the crest of the right ilium, while the integuments move freely over the tumor, are the prominent and least variable symptoms of this disease. The part so affected varies considerably in extent. When the swelling reaches across the abdomen towards the linea alba, or upwards obliquely towards the umbilicus, the disease should be suspected of having passed into an inflammation of the peritoneum, or even perhaps of the coats of the bowels. Pain of the upper part of the thigh in front, increased on motion, is either not a constant symptom, or is so trivial in the majority of cases, as to have escaped notice. The functions of the bowels are in general more or less affected. Nausea, vomiting, and anorexia are sometimes among the symptoms, but by no means essential to the disease. Symptomatic fever is not in general well-developed: in simple cases the pulse is almost natural; in the severer, it is sharp, hard, or jarring, not often small or oppressed. The disease is apt to come on insidiously; the colic pains, which usually usher it in, may last only for a few hours, or may recur at intervals for several weeks, before the swelling appears. The disease is of uncertain duration. When the precursory period is short, when it is itself uncomplicated, and the treatment appropriate, a cure may be expected in ten or twelve days—a degree of induration, however, generally remains. There exists sometimes a disposition to recurrence. The unfavourable terminations of this disease are suppuration and extension of the inflammation to the peritoneal lining of the abdomen, and even to the coats of the intestines. Resolution is the more common result. Even when it terminates by suppuration, as in the case of abscess of the pelvis after parturition, it is for the most part unattended with serious consequences. To pelvic inflammation after parturition the disease in question bears considerable analogy. Yet active treatment in the early stage is more essential than in the pelvic inflammation—for the tendency to suppuration seems to be proportioned to the neglect of free evacuations of blood. It seems certain that the pus formed in perityphlitic inflammation, sometimes, though rarely, penetrates through the abdominal wall. Such an occurrence, however, is extremely rare.

Dupuytren mentions the case of a young man who had been treated at the Orleans Hospital for a phlegmonous tumor in the right iliac region, which he at first neglected. He passed blood by stool, and his health was partially restored. He then went to Paris, where his complaint increased, the swelling enlarged, and abscesses opened in the right iliac region of a fistulous nature, through which pus and feculent matters were discharged. After a tedious illness of several months, accompanied with cough, diarrhœa, emaciation, &c. he recovered.

In the cases which proved fatal, no evacuation of pus either by the rectum or by an external opening for the most part takes place; but a copious deposit of pus is found on dissection almost uniformly, as far as the reported cases show, along with marks of extension of the inflammation to the peritoneum. Albers and others relate several cases of this disease, and record the several less favourable terminations of it to be; 1, Suppuration with discharge of pus by stool—2, Suppuration with discharge by an external aperture; and 3, Suppuration with superadded peritonitis. Dr. Seller relates a case which well illustrates the insidious mode in which this inflammation makes its attack. The patient was a medical man about 40. Towards the end of July, 1840, being apparently in perfect health, he awoke in the middle of the night with severe griping pain, which continued for two or three hours, and then passed off with a slight evacuation of the bowels. On getting up he felt nothing but a sense of weariness, and of weight and slight tenderness in the abdomen, as if the parietes did not sufficiently support the bowels—he went on for two days without any perceptible aggravation. On the evening of the third day, he noticed a slight pain in the fore-part of the right thigh, which he found to commence in the right iliac region. Next morning considerable pain on pressure in that part—a hard circumscribed swelling, with a dull sound on percussion—bowels free. Constitutional symptoms not well marked, pulse rather frequent, and harsh. Twenty leeches to the swelling with relief—venæsection in the evening to 16 ounces. At night a calomel and opium pill. Next day symptoms abated. Venesection and leeches again. Two days after more leeches, in consequence of some stinging pains in the part. After ten days from the commencement the patient walked out perfectly recovered.

The pathological seat of this affection seems to be in the cellular tissue between the fasciæ of the iliacus internus and coats of the cæcum. This explains why the pus, when produced, so uniformly passes into the cæcum. The iliac fascia, though somewhat variable in character, is for the most part a strong fibrous membrane. Its extent and connexions form, so long as it is free from disease, an impenetrable barrier to the progress of pus outwards. And when we remember that about one third part of the circumference of the cæcum is uncovered by peritoneum, and that the uncovered part is in contact with loose cellular tissue interposed between the iliac fascia and itself, we shall no longer feel any difficulties in understanding the chief peculiarities of peri-cœcal inflammation. The pus is confined on every side by the close adherence of the peritoneum to the cavity which it lines. The alternative is, on the one hand, that pus so confined, almost in a sac, should penetrate through the posterior wall of the cæcum, which is but making its way through a thin partition to a mucous cavity, or, on the other, that it should penetrate through the substance of the iliac fascia, or ascend into the right meso-colon by separating its laminæ, or detaching the peritoneum from the iliac fascia, where it adheres close to it, should make its way into the pelvis. Cases may be instanced in which each of the consequences under the latter alternative has occurred, yet very rarely by comparison, and not probably till the inflammation unchecked had extended beyond its original seat, and destroyed the substance of the fascia, or weakened the cohesion between the laminæ of the mesocolon, or that between the peritoneum and the subjacent substance.

---

### OVARIAN TUMOR.

In the Montreal Medical Gazette (April, 1844), is related a case of ovarian tumor bursting into the bowels with ultimate recovery.

A married female, aged 27 years, was delivered of a child in May 1842. On the 22nd November following, Dr. Fraser was sent for, and found her labouring under severe pain in the right iliac and lumbar region, shooting down to the thigh, with dysury. The pain abated; but soon returned. There was fulness in the right iliac fossa, with tenderness to the touch, and febrile symptoms. Leeching and purging rendered the fulness less conspicuous. On the 10th Dec. there was a severe attack of pain, and half-way between the pubis and umbilicus was discovered a well defined tumor, the size of a turkey's egg. There was no swelling or hardness in the original site of pain, and the present tumor was capable of being moved about, even beyond the mesian line. In four days it became the size of a cocoa-nut, with great pain and sense of distention. On the 14th Dec. an exploratory needle was introduced, and three ounces of a strawcoloured fluid evacuated, forming, when cold, a white jelly, when treated with nitric acid. The feeling of distention was relieved for some days, when it returned, and continued for six weeks, with varying paroxysms. The general health had now suffered much, and she became affected with dry cough. The tumor progressed in size, and on the 10th of January, 1844, it occupied a large space, pressing so much on the vagina, that no satisfactory examination could be made. On the 25th Jan. premature labour came on, and she was delivered of a fœtus about four months old. The pain still continued as severe as ever. Feb. 9th, a consultation was called; but the tumor, in the mean time, had burst into the bowels, from which a large quantity of dark, red, and fætid matter was discharged, the discharge continuing for three or four weeks, gradually decreasing, and at length entirely ceasing. The remains of the contracted sac are still to be felt in the lower part of the abdomen. The general health has improved: but the sequel is far from certain. We have some doubts about the precise nature of the disease.

---

### Antimony in Infants. (Provincial Journal, July 1, 1844.)

Mr. Wilton (Surgeon to the Gloucester Infirmary) has drawn the attention of the profession to the serious effects which sometimes result from the exhibition of antimony in infantile diseases. The first case related was that of a child about a year old, to whom the mother had given small doses of antimonial wine for a cold and affection of the chest. Mr. W. found the patient suffering from slight convulsions—pallid sunken countenance—vomiting and diarrhœa. The means employed failed, and the child died. On dissection, the internal and external parts were pale and exsangueous—no vascular patches in the alimentary canal—the brain very soft; but no organic changes anywhere to account for the sudden collapse and death.

A few days afterwards, Mr. W. was summoned to another child, exhibiting similar symptoms, after taking antimonial wine. This child was saved. Some other cases are narrated, of which we shall notice the following. A child, about four years of age, became affected by cold, cough, and febrile symptoms. Saline draughts with antimonial wine were given by the medical attendant. Sickness and diarrhœa followed, with sudden prostration, which ended in death, despite of cordials and stimulants. On dissection, the surface of the body was pallid, and on being moved, a large quantity of colourless fluid flowed from the mouth. The whole body was exsangueous, but no marks of inflammation were anywhere visible.

We have often seen distressing symptoms follow antimony in children, and, except in urgent or croupy cases, we generally prefer ipecacuan, which, if it sickens at all, soon clears itself out of the stomach without injury. Mr. Noble, of Manchester has also published some precautionary remarks in the same Journal on antimony in children, which are worthy the perusal of all junior practitioners.

---

### Eupatorium Perfoliatum in Epidemic Influenza. By J. F. Peebles, M.D. Petersburg. (American Journal of the Medical Sciences, April, 1844.)

To this herb the domestic name of Boneset was given, from its prompt manner of relieving pains in the limbs and general muscular system, which attended a peculiar form of febrile disease which prevailed many years ago in the northern parts of this country (America). This fact led to the suggestion of its employment in epidemic influenza. It was found that the pain in the back and limbs, and the lassitude of the general muscular system subsided as soon as the system was placed under its influence. Besides this prompt action on the nervous system, the eupatorium perfoliatum united in its operation other qualities, each one eminently adapted to fulfil some important indication in the treatment of the influenza. Among the first of these may be mentioned its diaphoretic powers. In this disease the skin was not unfrequently imbued with perspiration; the perspiration, however, was of a morbid character, resulting apparently from a lax condition of the skin, which under such circumstances was always pale and morbidly sensitive. Antimonials were ineffectual in removing this state—the opium in the pulvis Doveri checked the other secretions, and was therefore objectionable. Entirely exempt from this objection the Eupatorium not only induced a healthy and free perspiration, but quickly restored the skin to its natural and healthy state, rendering its texture firm and healthy. Under its use also the disposition to cough subsided, and there was an immediate amelioration of all the pulmonary symptoms. It also proved to be a prompt and efficacious *expectorant*. It was also found to possess tonic properties peculiarly suited to the treatment of certain cases of epidemic influenza in aged persons.

*Manner of Administration.*—Where it was determined to treat the disease with the herb alone, the patient after being covered in bed was made to swallow a wine-glass of the infusion, prepared by infusing one ounce of the dried leaves in a pint of boiling water, warm, every half-hour. After the fourth or fifth dose considerable nausea, sometimes vomiting, with free diaphoresis ensued, and there was an immediate amelioration of all the symptoms. Expectoration also was produced. After this relief ensued, and the patient felt very comfortable. The infusion was now given only every third or fourth hour in the same dose. The bowels were generally opened in about six hours after the commencement of the treatment, and remained in a lax condition.

---

### OVARIOTOMY. DR. CHURCHILL.

Ovariotomy is, just now, a formidable rival for fame, with Mesmerism or Hydropathy. The two former, indeed, ought to go hand-in-hand; for as ovarian tumors seldom grow in any but the patrons and recipients of animal magnetism, it would be a great advantage to those who come under the scalpel, to have its pains and penalties annihilated by the passes of an adroit mesmerist. Be this as it may, the ovarian operation can be tested only by time and statistics—the advocates and opponents steering such opposite courses, and using such ingenious arguments, as to puzzle the practitioner. Statistics will settle the question. Dr. Montgomery has gone into considerable detail on this point, and collected from various points of the compass a mass of materials that may greatly assist our prognosis—perhaps even our diagnosis, in these dangerous cases.

Dr. M. properly remarks that, under the head of ovarian dropsy, are comprehended many swellings very different from dropsy. There may be a single— or many cysts—and the contents of the cysts may and do vary from clear serum to an almost wholly solid substance. The ovaries may consist of malignant deposits—and last, not least, they may be detached, or they may have acquired extensive adhesions to various adjacent parts, rendering a successful operation all but impossible.

Mr. Southam has published the result of 20 cases of paracentesis—ten from Bright—five from Barlow—and five of his own. Out of these 14 died within nine months after the first operation. Of the remaining six, two died in eighteen months—and four lived for several years, from four to nine.

Of eleven cases of ovarian dropsy admitted into Guy's Hospital, seven were tapped, three of which were unsuccessful. The proposal of injecting stimulating fluids into the emptied sacs, has, we believe, either never been tried, or entirely abandoned.

The following three tables will exhibit a coup d'œil of the results of almost all the cases on record. It has been constructed with great care and labour by the able and indefatigable author.

TABLE I.—*Cases of Extirpation of the Ovary.*

| No. and Date. | Operator. | Age | Incision. | Result. | Character of Disease. | Adhesions. |
|---|---|---|---|---|---|---|
| 1 | L'Aumonier. | .. | 4 inches. | Recovered. | Abscess of ovary. | |
| 2—1809 | Dr. M'Dowal. | .. | 9 do. | do. | Gelatinous matter. | |
| 3—1816 | do. | .. | Long. | do. | Scirrhous ovary. | |
| 4 | do. | .. | .. .. .. .. | do | | |
| 5 | do. | .. | .. .. .. .. | do. | | |
| 6 | do. | .. | .. .. .. .. | Died. | | |
| 7—1821 | Dr. N. Smith. | 33 | 3 inches. | Recovered. | Cyst, fluid. | Adhesions. |
| 8—1825 | Mr. Lizars. | 36 | Long. | do. | | |
| 9—1825 | do. | 35 | do. | Died. | .. .. .. .. .. | Adherent. |
| 10 | Dr. A. G. Smith. | 30 | do. | Recovered. | Cyst, fluid. | |
| 11 | Dr. Quittenbaum | .. | About 4 in. | do. | | |
| 12—1829 | Mr. D. Rogers. | .. | About 3 in. | do. | Solid and fluid. | Adhesions. |
| 13 | Dr. Granville. | .. | .. .. .. .. | Died. | | |
| 14 | Dr. Chrysmer. | 47 | Long. | do. | Cart. and lardaceous matter. | Adherent. |
| 15 | do. | 38 | do. | Recovered. | Honey-like and green sanies. | do. |
| 16 | do. | .. | do. | Died. | | |
| 17 | Dr. Ritter. | 31 | do. | Recovered. | Cyst, fluid. | |
| 18—1836 | Mr. King. | 57 | Short. | do. | do. | |
| 19—1833 | Mr. Jeafferson. | .. | do. | do. | do. | |
| 20 | M. Dolhoff. | 23 | Long. | Died. | Cyst and fluid. | Adhesions. |
| 21—1836 | Mr. West. | .. | Short. | Recovered. | do. | |
| 22 | do. | .. | do. | do. | do. | |
| 23 | do. | 24 | do. | Died. | do. | |
| 24 | do. | .. | do. | Not cured. | do. | |
| 25 | Mr. Hargraves. | 40 | do. | do. | Multiloc. cysts. | Adhesions. |
| 26 | Dr. Clay. | 46 | 27 inches. | Recovered. | Cysts, sol. and fluid. | do. |
| 27 | .. .. .. .. | 67 | 14 do. | do. | do. | Ext. adh. |
| 28 | .. .. .. .. | 39 | 28 do. | do. | do. | do. |
| 29 | .. .. .. .. | 40 | 14 do. | Died. | do. | do. |
| 30 | .. .. .. .. | 22 | 14 do. | Recovered. | do. | Adhesions. |
| 31 | .. .. .. .. | 40 | 14 do. | Died. | do. | None. |
| 32 | .. .. .. .. | 43 | 14 do. | Recovered. | do. | Ext. adh. |
| 33 | .. .. .. .. | 59 | 16 do. | Died | do. | do. |
| 34 | .. .. .. .. | 46 | 16 do. | Recovered. | do. | do. |
| 35—1840 | Mr. B. Philips. | .. | 2 inches. | Died. | | |
| 36—1841 | Dr. Stilling. | .. | 6 do. | do. | | |
| 37—1842 | Mr. Walne. | 58 | Long. | Recovered. | do. | None. |
| 38—1843 | do. | 57 | do. | do. | do. | do. |
| 39 | do. | 21 | do. | Died. | | |
| 40—1843 | do. | 20 | do. | Recovered. | do. | do. |
| 41—1843 | Mr. Morris. | .. | do. | do. | | |
| 42—1843 | Mr. Southam. | .. | do. | do. | Cystic sarcoma. | do. |
| 43—1843 | Dr. F. Bird. | .. | 3 or 4 in. | do. | Cyst and fluid. | do. |
| 44—1844 | do. | .. | do. | do. | Cysts and solid matter. | do. |
| 45 | Mr. Atlee. | .. | 3 inches. | do. | .. .. .. .. .. | Adhesions. |
| 46 | Mr. Lane. | .. | Long. | do. | Cysts, fluid. | None. |
| 47 | Mr. Key. | 19 | do. | Died. | do. | do. |
| 48 | Mr. Greenhow. | 29 | do. | do. | .. .. .. .. | do. |
| 49 | Mr. B. Cooper. | 32 | do. | do. | | |

TABLE II.—*Cases of Ovarian Disease in which the Operation could not be completed.*

| Date. | Operator. | Cause of Failure. | Result. | Incision. |
|---|---|---|---|---|
| 50 | Dr. M'Dowal. | Adhesions to bladder and uterus. | Recovered. | Long. |
| 51 | Mr. Lizars. | Solid and very vascular tumour. | do. | do. |
| 52—1826 | Dr. Granville. | Firm adhesions. | do. | 6 inches. |
| 53 | Dr. Dieffenbach. | Vascularity. | do. | Long. |
| 54—1826 | Dr. Martini. | Solid and fixed tumour. | Died. | do. |
| 55 | Anonymous. | Fixed tumour. | do. | |
| 56 | M. Dolhoff. | do. | do. | About 6 in. |
| 57 | Dr. Clay. | Exten. adhesions. | do. | Long. |
| 58 | Mr. Walne. | do. | Recovered. | 5 inches. |

TABLE III.—*Cases in which the Operation failed from an Error in Diagnosis.*

| Date. | Operator. | Result. | Disease. |
|---|---|---|---|
| 59—1823 | Mr. Lizars. | Recovered. | No tumour found. |
| 60—1834 | Mr. King. | do. | do. |
| 61 | M. Dolhoff. | do. | do. |
| 62 | Dr. Clay. | Died. | Uterine tumour. |
| 63 | do. | Recovered. | Hydatid. |
| 64 | do. | Died. | Pelvic tumour. |
| 65 | do. | do. | Uterine tumour. |
| 66 | Mr. Heath. | do. | do. |

Thus, the entire number amounts to 66, of which 42 recovered and 24 died—or about 1 in 2½. Of the 49 cases in which the ovary was extirpated, 16 died, or 1 in 3. Of the nine cases in which the operation could not be completed, four died—or 1 in 2¼; and of the eight cases where the operation was unnecessary, 4 died, or 1 in 2.

Age does not appear to have had much influence, beneficial or otherwise, and the same may be said of marriage. Adhesions render the result of the operation much more dangerous than freedom from the same, and yet not so much so as one would, *a priori*, expect. Where other organic diseases co-existed with ovarian, the termination was almost always fatal. It is strange that the operation should have been ever performed, where no tumor has existed; yet the mistake has been made by eminent surgeons, and without any negligence on their parts. Dr. Montgomery mentions a case where he felt a distinct tumor in a female's abdomen, which suddenly vanished in the very act of examination! The abdominal muscles, in fact, often act in such a way as to imitate organic enlargements of the liver, spleen, ovaries, &c., and thus deceive even the most careful practitioners. After many judicious remarks, cautions, and comparisons, our author comes to the following conclusions:—

"Even after the details I have given, it is very difficult to come to a definite, and perfectly satisfactory conclusion, because, 1, we have not sufficiently accurate data to estimate the progress of the disease unaided by surgery. 2. The table quoted from Mr. Southam is clearly too limited to afford a fair average of the results of tapping, and it is not easy to obtain sufficient facts to enlarge it. 3. The cases in which ovariotomy has been performed are of such a mixed character, that it is impossible to select with fairness those cases in which the operation was demanded for the relief of urgent suffering, and suitable to the nature of the disease, without the appearance of partiality. And 4, from the obscurity of the diagnosis, it is too much, perhaps, to expect that our practice in future will be free from those drawbacks on the operation.

"But bearing in mind these difficulties, and making allowance for those drawbacks, I think we may conclude that there are cases in which the operation would be justifiable; and on these grounds,—we find the general opinion is against the curability of the disease by medical means;—that after a time the patient will die from local disease or accident, or constitutional disturbance, and that meantime she suffers more or less inconvenience;—that tapping in almost all cases affords but temporary relief;—and that, as far as the limited statistics we have adduced are admissible as evidence, it is attended with great danger: i. e. 1 in 5 died of the first operation, and of twenty patients, fourteen (more than two-thirds) died within nine months of the first tapping; whilst of the entire number of those who underwent the operation of ovariotomy, about one-half have absolutely recovered so far."

The foregoing paper is very creditable to the industry, the talents, and the judgment of its author.—*Dublin Journal, July* 1844.

---

### Empyema—Perforation of Lung, &c.

Mr. Harrison, of Reading, has related a case of this kind in our Provincial contemporary, of which we shall give an abstract.

Sarah Lukeman, aged 50, was admitted at the Reading Dispensary, July 3, 1843, after nine weeks illness. Severe rigors had been followed by pain in left side, with fever, cough, and some expectoration. At present the left side contracted, and fixed in respiration, with universal dulness on percussion there. Respiration, anteriorly and latterly, distant and audible, with large crepitation in the scapular region. Loud vocal fremitus—puerile respiration in the opposite lung. Lies on the right side—cough frequent—expectoration profuse and purulent. *Diagnosis.*—Pleuro-pneumonia with effusion. Calomel and Dover's powder, with hydriodate of potash. She was admitted into hospital under Cowan, and remained there three weeks, without benefit, when she was discharged at her own request. She was re-admitted, under Dr. Harrison on the 31st July. The contraction of left side was still advancing—gurgling crepitation at apex of lung —expectoration profuse. The apex of right lung also was dull. Acetate of lead with opium every six hours; with good food and wine. Under this treatment she greatly improved.

*Aug.* 11. Yesterday felt as if something had given way in her chest, and she immediately expectorated a tea-cupful or more of nauseous purulent fluid. She is now much relieved, and the cough and expectoration, since yesterday, but trifling. The heart's pulsations are now more distinct at the apex of left lung, than in the ordinary place. For some days she was so well as to go down stairs, and even walk in the garden. On the evening of the 14th of August, she suddenly experienced a sense of suffocation, and brought up about half a pint of the nauseous matter. Frightful dyspnœa continued, with some mitigation, till her death, seventeen days afterwards. During that period, she daily expectorated a large quantity of the above-mentioned fluid.

*Post-mortem.*—On opening the chest, the right lung was seen projecting beyond the median line—the left was shrunk and hidden, and the cavity closed by adhesions of the pleura to the pericardium. When the cavity was opened, there was about a pint of thick purulent fluid in it. The lung was compressed to the vertebræ, and to the diaphragm. The costal pleura was lined by an adventitious substance, partially osseous, and presenting a worm-eaten appearance. On separating the osseous layer, several small pouches or cavities were disclosed—the extremities of enlarged bronchial tubes. The lung was of a dark grey colour, soft, and, at its apex, in a state of incipient gangrene. The bronchial tubes were enlarged, and the lung through them, had received air throughout, till they were filled with purulent infiltration. There was an aperture of communication with the left bronchus. Right lung large, spongy, and free from congestion. No tubercles in either of the lungs. Heart small and feeble—pericardium and endocardium healthy.

These are the naked facts of the case, to which Mr. Harrison has appended numerous comments and quotations, for which we must refer to the Provincial Journal for June 26, 1844.

---

ON THE PULSE OF THE INSANE. By PLINY EARLE, M.D. (From the American Journal of the Medical Sciences, April 1844.)

It is but seldom that allusion is made to the state of the pulse in the writings of those who have treated of mental alienation. This arises from the generally admitted fact, that the state of the pulse is a less certain index of the true nature of the disease, of its mildness or intensity, than it is in other affections. To this general truth there are however many exceptions, of which the following is one: " whenever it (the pulse) exhibits very considerable changes, without any obvious causes or corresponding symptoms, sudden death frequently closes the scene." In phrenitis accompanied with delirium, the pulse is frequently contracted, small and apparently feeble. Under these circumstances it almost invariably becomes fuller and stronger after the abstraction of blood from the arm. Dr. Spurzheim barely alludes to this condition when speaking of the treatment of cases in which there is a hypersthenic state of the brain. In patients of a nervous temperament, where great irritability is present, the pulse may be not only unnaturally frequent but abnormally developed, full and hard. Yet were the physician to be influenced by this sign and to resort to venæsection, he would in most cases only aggravate the disease. It has been observed, as a peculiarity worthy of attention, that the pulse differs in the radial and carotid arteries, and, when soft and weak in the former, is full and hard in the latter, though the number of pulsations in the minute be the same in both. In cases where this is observed, there is generally an abnormal determination of blood to the brain, with flushing of the face, preternatural heat of the head, and coldness of the extremities. The pulse in some maniacs is affected by the most trifling causes; in others it suffers little variation, however violent the attendant symptoms. With respect to the value of the pulse, as a guide in cases of insanity, Dr. Rush speaks as follows: " should all the above marks fail of deciding the state of the mind as to sanity, recourse should be had to the state of the pulse. It is, with but few exceptions, more frequent in all grades of madness than in health. In " melancholy madness," says Van Swieten, " the pulse beats slower and the body feels colder." Esquirol, in reference to the same type of disease, says, " the pulse is ordinarily slow, feeble, concentrated; sometimes it is very hard, and a kind of fremissement of the artery is felt under the finger." The same author says, " among the insane, some are plethoric, others lymphatic, some strong, others feeble; in the former, the pulse is full, hard and developed, in the latter, slow, soft and concentrated." From a variety of statements and experiments made and instituted by the writer of this Essay, corroborated also by those of others, he comes to the general conclusion, that the pulse of lunatics is more rapid than that of the sane. When, however, it is recollected that the circulation differs greatly in different persons, even in health, and that there is a vast diversity of influences, which, in health or disease, may determine the acceleration or diminution of its rapidity, but little value can be placed upon the pulse as a test in insanity.

---

### Hydrocele.

Dr. Porter, of the Meath Hospital, Dublin, has published some Practical Commentaries on the various Operations for Hydrocele, and has tendered his own to the profession, through our Dublin contemporary for July last.

"This operation is partly that by incision, the only difference being, that instead of dividing the tunica vaginalis in the entire extent of the tumour, my incision extends only from an inch to an inch and a half in length: and partly that by the tent, an operation first proposed (it is said) by Franco, but revived and recommended by the celebrated Larrey. Having first punctured the tumour in order to examine the state of the parts, and satisfy myself that it is a case in which an attempt to cure the disease radically may be safely made, or at least in which such attempt would be justifiable, I allow the sac to fill again. When the disease has re-appeared, and the tunica vaginalis is as much distended as it previously had been, I perform the operation thus: Having that part of the scrotum in which I intend to operate shaved, I make the incision of the length above mentioned, down to the tunica vaginalis, and examine carefully whether any vessel has been wounded that could possibly furnish a considerable quantity of blood. I then pass a bistoury into the tunica vaginalis at one extremity of the incision, out at the other, and divide it by a rapid withdrawal of the instrument. Having completed the incision, a tent of rolled lint, moistened with oil, and secured with a ligature, so as to be easily withdrawn, is introduced. The operation is then completed. The patient may be placed in bed. On the succeeding day I generally bleed from the arm to the extent of ten, twelve, or fourteen ounces, and particularly if the scrotum is red, and shews a tendency to inflammation. Latterly I have adopted this practice as a preventive in all cases with apparently the most satisfactory results. The tent is left to become loose, and drop out of itself, which usually takes place on the third or fourth day, and need not be replaced; but it is desirable to break up any adhesions that may be formed between the lips of the wound, and to introduce the finger occasionally into the cavity of the tunica vaginalis until the sixth, after which it may be treated with light superficial dressing, and the cure is generally perfect in about three weeks."

Dr. Porter has now practised this operation for fifteen years, and comparing it with others, has no reason to feel dissatisfied. It is scarcely more painful, he avers, than the ordinary puncture of the common trocar; and, if carefully performed, is free from the possible occurrence of any untoward accident. Future experience must determine whether the tendency to relapse is greater after this than after other operations which are termed radical.

---

Two Cases of Icthyosis Intra-Uterine. By J. Y. Simpson, M.D. F.R.S.E. &c. &c. (London and Edinburgh Monthly Journal of Medical Science, July 1844.)

*Case* 1.—This case was communicated to Dr. Simpson of Edinburgh, by Professor

Vrolik of Amsterdam. The child was a male, and born in the eighth month—parents perfectly healthy. The skin of the infant, fissured in different parts, was hard and thickened generally: it was of a yellow colour, and covered with the epidermis; in the fissures it was softer, and somewhat of a bloody appearance. The head, which was almost round, presented very open sutures, and a very large anterior fontanelle. It was covered with thin, short, silky hair, and the scalp was intersected by numerous fissures, one of them running from the left superior eyelid over the os frontis, being twelve millimetres in breadth. Another was confined to the right superior eye-lid, and here also there was a very large fissure at the external angle of the eye-lids, which ran in a direction backwards. The forehead was inclined backwards; the ears were quite concealed by the skin, and only to be seen in their outline very imperfectly; the eye-brows were entirely absent; instead of closed eye-lids, there were two bloody tumors formed by the interior lining membrane of the eye-lids, or by the ectropia of the conjunctiva, behind which the eye-balls were deeply concealed in the orbits; there were but few cilia on the eye-lids, and the extroversion of the conjunctiva was less in the under than in the upper eye-lids. Between the two very distant eyes there was some indication of a nose, which was very large and flattened, and indeed only shewn to be such by the two nostrils. The mouth was largely opened, forming an ellipsis with the returned interior surface of the lips, and surrounding the alveolar parts of the two jaws and the tongue, which was much thickened; in the upper jaw the rudiments of the incisor teeth were very apparent: at each angle of the lips there were three cutaneous fissures; and in the same manner there was one on each side of the upper lip. Neck short and broad; breast and abdomen were almost of the same size, and very different from what they are under normal circumstances; the upper and lower extremities were so far natural, but the hands and feet were swollen, and covered with a fissured skin, and the fingers and toes resembled claws. The scrotum (containing no testes) and the penis were of a red colour, and appeared as if the chorion and epidermis were retracted from them; no perinæum was to be seen; the stretched skin did not allow of a depression behind the scrotum; the opening of the anus was placed on the same level with the nates; here, as well as on the thighs, there were a great many fissures, which, by some difference in their appearance, shewed that they had not all been formed at the same time. It was evident that some of these fissures were recent and unhealed; others were in the way of being cured; some were covered by a red cicatrix; and others were quite like the unfissured parts of the skin. Dr. Vrolik coincides in a great degree with Dr. Simpson regarding the etiology of this deformity, viz.: that the cutaneous fissures are merely a secondary and a mechanical result, and that the integumentary parts not possessing a proper degree of expansibility, and not increasing with the increasing dimensions of the growing fœtus, at last split and fissured under the stretching from within to which they are subjected. To the same mechanical cause are to be referred the ectropia of the eyelids, the form of the mouth, of the nose, and of the ears. The only part in which his explanation differs from that of Professor Simpson is, that the general form of the child must depend upon an arrest of development, and that both this and the degeneration of the integuments are the causes of this deformity. The other case of this disease occurred at Haddington, and bore an exact similitude to the one just described, except that in the Haddington case the disease was confined to the head and neck—in the latter case also the fissures were deeper and wider. In this case also the child was enormously large—it had been born at the full time. It appeared viable enough—it died, however, in a few days.

---

Observations on the Value of the Chemical Tests of Diabetic Urine. By W. T. Gairdner, Esq. Edinburgh. (London and Edinburgh Monthly Journal of Medical Science, July 1844.)

Dr. Golding Bird has described in the Medical Gazette the most notable of the tests which have been proposed for diabetic urine.

1st. *Hunefield's test,* founded on the power of sugar to deoxidate chromic acid—this is considered fallacious.

2nd. *Runge's test,* which consists in the development of a black colour by a drop of sulphuric acid.

3rd. *Trommer's test*—to be described presently.

4th. *The fermentation test.*—Diabetic urine treated with yeast, and left for a few hours in a warm place, becomes troubled, and covered with a frothy scum; at the same time it disengages carbonic acid gas, which being collected and measured, indicates the quantity of sugar, one cubic inch corresponding nearly to a grain of sugar.

5th. *The microscopic test,* which depends on the development in urine impregnated with sugar, of minute ovoid particles, the spores of a confervoid or fungoid vegetation which attends on the vinous fermentation in all saccharine fluids.

The fermentation test is not liable to any obvious source of fallacy, and it is one of peculiar excellence in this respect, that it furnishes us with a very accurate and simple means of estimating the amount of the sugar, if necessary.

Runge's test is best performed in the following manner; the urine should be evaporated on a white plate over a vapour bath, or carefully with very gentle heat, as it is difficult to avoid charring, if the proportion of sugar be considerable. A drop of the acid, diluted with six parts of water, should next be applied; and then by moving the plate two or three times backwards and forwards over the flame of a spirit lamp, the black hue is brought out in a spot with clearly defined edges, if there be any sugar present.

Trommer's test is described as follows:—" Add to the suspected urine, contained in a large test-tube, a few drops of a solution of sulphate of copper; a very inconsiderable troubling generally results, probably from the deposition of a little phosphate of copper. Sufficient *liquor potassæ* should then be added to render the whole strongly alkaline: a greyish-green precipitate of hydrated oxide falls, which if sugar be present, wholly or partially re-dissolves in an excess of the solution of potash, forming a blue liquid, not unlike the blue ammoniuret of copper. On gently heating the mixture nearly to ebullition, the copper falls in the state of suboxide, forming a red and copious precipitate. *If sugar is not present, the copper is deposited in the form of black oxide.*"—(*See Dr. Bird's paper.*)

Mr. Gairdner repeated this test with healthy urine, as well as with diabetic urine, and found that, in two specimens of perfectly healthy urine, a precipitate was obtained by Trommer's process, which in colour and appearance was entirely different from the black oxide of copper. The precipitates in question were in rather smaller quantity than usual, flocculent, and of a light orange colour, and were surrounded by a fluid deeply tinged with orange. On adding sugar to a portion of one of those urines, and subjecting it again to the test, the precipitate which resulted differed in no perceptible degree from the former. The precipitate in the diabetic urine operated on (which was very strongly saccharine) differed from these in being much more abundant, and somewhat brighter in hue; but the colour was essentially the same in both, viz. deep yellowish-orange. The fallacious nature of the test is clearly indicated by these observations; other experiments have still further confirmed Mr. Gairdner in this idea, and incline him to the conclusion that it is not the sugar alone which is concerned in the changes in diabetic urine. The tendency of sulphate of copper to form peculiar compounds with organic principles of all kinds, and the extremely various results when such compounds are submitted to Trommer's process, is another circumstance which tends to induce a suspicion of the fallaciousness of this test. Thus, a dilute solution of albumen (white of egg) gives a dark brown precipitate when treated by Trommer's process; a single drop or two of tincture of galls gives a red or purple precipitate in a brownish liquid; and a greatly diluted portion of skimmed milk gives a gold-coloured precipitate in a bright green liquid. Mr. Gairdner deems it probable that the precipitate thrown down from diabetic urine by Trommer's test is not uncombined protoxide of copper, but that both in this case and frequently in healthy urine, that precipitate is the result of a combination of the oxides of copper with various animal matters which occur in the urine.

---

REMARKS UPON THE EARLY CONDITION, AND PROBABLE ORIGIN OF DOUBLE MONSTERS. By ALLEN THOMSON, M.D. F.R.S.E. &c. &c. (London and Edinburgh Monthly Journal of Medical Science, July 1844.)

In the preceding Number of the Journal we gave an analysis of the first part of Dr. A. Thomson's Paper on the subject of double malformation. We shall now present to our readers a few remarks from the same writer upon the theories which have been proposed to explain their origin. Three speculative views have been taken of this subject. 1. The double monster has been supposed to have proceeded from two distinct germs, which have become united in the course of development; 2. It has been held to have originated in a single germ, which has become double or has been subdivided; and 3. The germ has been regarded as abnormally compound from the first, implying that the organs and parts composing the double monster are at once produced from the germ, without either separation or coalition of its parts, other than belong to the natural process of development. This last view appears to be more in accordance with our knowledge of embryology than either of the two before mentioned.

The various forms of double mal-formation, considered with a reference to the theory of their origin, may be distinguished into two sets; in one of which the united embryos, or, if they are not complete, the parts composing them, have arrived at a nearly equal degree of development; in the other, an imperfectly formed body, or a part of one, is either attached to or inclosed in a more perfect embryo. This last set includes the various forms of Parasitical and Inclosed Double Malformations. The following arguments are among the more convincing that may be adduced in support of the view that double monsters are of original formation, and not the result of any secondary or accidental separation or combination of germs.

1. The different varieties of double monsters form together a complete series of gradation, from the smallest to the greatest degree of the double condition; and in this series, as in those of simple mal-formations, the forms are by no means infinitely varied, but analogous deviations of structure are constantly returning; so as to give no countenance to the idea, that any variation of external circumstances could determine either the manner or the degree of combination existing between the two component individuals.

2. In this combination of the two individuals, or of some of their parts, the most remarkable symmetry is preserved: for there is not any appearance of the junction of parts fortuitously brought into juxta-position; but, on the contrary, an invariable union of only similar or corresponding parts in the two bodies. For the most part that union is to an equal extent in the two individuals; and in the majority of those cases wherein the duplicity is a mere redundance, this superadded part is not single or unilateral, but rather median and common.

3. We observe in twin conceptions the ova frequently pressed closely together, without any tendency to the union of the fœtuses. These circumstances render it improbable that mere accident can have contributed to any great degree to bring about the union of the parts of the two embryos.

Still, however, though it appears necessary that the compound parts of a double monster should be at once formed and developed as such from the monstrous germ in which they originate, it is obvious that the process of double development must differ in some important respects from that in a single embryo. It is interesting to observe here that while the development of the parts on the external or remote sides of the two united embryoes may be quite or nearly natural, that of the uniting parts must generally be of an anomalous kind.

In the separate parts of double monsters, their development is independent, and may be natural,—in the united portions it is compound and abnormal.

Another subject of interest connected with the history of double monsters, is the relation between the symmetrical arrangement of the organs in the two united individuals, and the position of the embryos in the ovum at an early period. It is a remarkable fact that those internal organs of the body, such as the heart and liver, stomach, pancreas, colon, &c. which are non-symmetrical, are generally reversed in the body of one of the individuals composing a double monster. Von Baer offers the following theoretical explanation of the cause of its occurrence.

The embryo of the bird during the first thirty-six hours of its formation, lies with its abdominal surface downwards, and its dorsum upwards, and its parts are nearly in a symmetrical position; but in the course of the third day of incubation in the egg of the common fowl, a change of position occurs, by which first the head, then the thoracic part of the body, and still later the abdominal and caudale portions, come to be laid with the left side of the embryo towards the subjacent surface of the membranes of the yolk; so that in opening a fowl's egg, the small end being directed away from the observer, the cephalic extremity of the embryo will generally be found directed transversely across the yolk, laid in part on its left side, and with its head towards the left hand of the observer. Von Baer had remarked, in very rare instances in the bird's egg, more frequently in the ovum of the pig, the embryo lying with its right side applied to the yolk; and he has very ingeniously supposed that inversion, partial or general, in the position of the non-symmetrical organs, may be connected with this mal-position of the embryo at an early period.

The union between the two embryos composing a double monster may be either visceral or cerebro-spinal. The latter mode of union, when simple, is of comparatively rare occurrence—it in general is by more or less of the visceral parietes, that the two individuals are conjoined. The best known instances of cerebro-spinal union are those in which a part of the cerebro-spinal axis (upper or lower) is common to the embryos, and the remainder double. In such cases, however, there is also invariably visceral union to a considerable extent. A much rarer form of cerebro-spinal union occurs in those examples in which there is no visceral fusion, there being two complete or nearly complete embryos, conjoined either by the *vertices* of their heads, or by the opposite extremities of the vertebral column. In cases of lateral union of two embryos by the visceral parietes, there is generally more or less of the fusion of corresponding parts of the two embryos between them, such fusion being attributable to the simultaneous and compound development of the united parts. But there are instances of an intimate union of this kind occurring on both sides of the body in those double monsters in which the embryos are opposed to each other, more or less directly, face to face. We may here refer to the very remarkable symmetrical union of the parietes of the body or head, presented in the various forms of the Janus-like double monsters. We shall return to this interesting Paper at a future period.

### CHRONIC EMPYEMA—PARACENTESIS, &c.

Dr Wells, of Columbia, relates an interesting case which occurred in the person of a medical gentleman, about 33 years of age, when he came under his care in the beginning of June, 1836. About 12 months previously he had an intense pleurisy of the left side, which gave way to active treatment, leaving a pain and sense of weight in that side, with slow fever, quick pulse, dyspnœa, and inability to lie on the right side. In four or five weeks these symptoms partially subsided, and his appetite returned; but dyspnœa and palpitation remained. Fluctuation became evident in the left cavity of the pleura, and the ribs of that side were bulged out.

*June*, 1836.—The heart was found beating in the right side of the chest—no respiratory murmur in the opposite side, which was one-third larger than the right—intercostal spaces very wide—hectic fever—pulse small and rapid—face bloated, with various other symptoms indicative of the nature of the case. On the 7th June paracentesis thoracis was performed, and between two and three quarts of thin inodorous pus were discharged, with much relief, but with such faintness that the further issue of fluid was stopped. In the evening two quarts more of the fluid were drawn off—faintness was produced—and another interruption occurred. In the middle of next day, June 8th, the tube was introduced, and about two quarts of fluid were drawn off, when the stream was interrupted, the thoracic parietes not being capable of farther compression. 9th. Complained of oppression, and about a quart of fluid was abstracted, with relief. 10th. Another quart drawn off. A quantity of air was this time sucked in, and produced inconvenience. 11th. Febrile excitement—embarrassed respiration. Drew off a quart of intolerably fœtid matter. 12th. Less fever—similar discharge from the chest. 13th. Discharge the same—debility increasing. A quarter of a pint of weak solution of chloride of soda was injected into the chest, and the greater part of it soon afterwards withdrawn. 14th. Did not experience any inconvenience from the injection, but seemed relieved by it. The irritation and fever subsiding—fluid withdrawn this day less fœtid. The injection repeated, and most of it suffered to return, after a few minutes.

"It is unnecessary farther to detail the daily treatment in this case. It was found impracticable to prevent more or less air passing into the chest, at each operation, and as little or no inconvenience resulted from it, under the counteracting influence of the injection, it was no longer regarded. The free extremity of the elastic tube was bent downwards below the level of that within the cavity, giving to the instrument the properties of a syphon, and the whole of the secreted fluid daily evacuated, and the solution of chloride of soda injected, gradually increasing its strength and its *quantity*, until it corresponded with the *quantity of matter* drawn off at each operation. From this time to the 20th of July, when he left Columbia, the same course was followed up. The discharge soon lost its fetor, and gradually decreased in quantity from the time we commenced the use of the injection, so that on his departure it amounted to about three gills in the twenty-four hours.

"During this period, a little over a month, his recovery was uninterrupted by any untoward symptoms; the state of his digestive organs was carefully watched, under the influence of an alterative, tonic course; and as his appetite and powers of digestion improved, he was allowed a more liberal diet. For the last ten days he remained here he was able to take a short walk daily, and with much more ease to himself than when he came; the prominence of the left side of the thorax, and upper part of the abdomen, had subsided; the heart had made some progress towards the left; but no respiratory murmur was yet perceptible on that side. His appetite was good; sleep much improved; and when he left me for

Cheraw he felt full confidence of a speedy recovery. He was advised to continue the same course of treatment until his health should be restored."

The month of August was spent at a mineral spring in South Carolina, with considerable benefit to the patient's health.

On the 7th March, 1837, he reported that his health was progressively improving—that he had no cough, no pain, and was able to ride on horseback without fatigue more than 20 miles in a day. The quantity discharged now was about a gill in the 24 hours, being thin and inodorous. The left side of the chest had fallen in considerably. He had, for some time, left off the injection, and, indeed, all medicine. In March, 1844, Dr. Queen, his medical attendant, wrote to Dr. Wells to say that the fistulous opening still continued, and the discharge was nearly the same as in May 1837. The left side of the chest was very much contracted, and the right side bulged out. The heart, however, had nearly regained its natural position; but there is no respiratory murmur in the left side of the thorax. His general health was very good, and he was able to take considerable exercise without much fatigue. Dr. Wells has added to this case several judicious reflections on Paracentesis Thoracis and Empyema.—*American Journal of the Medical Sciences.*

---

### Phlegmasia Dolens in Males.

Mr. Greenhow relates (in the Prov. Journal) the case of a young man in the Infirmary of Newcastle-on-Tyne, who was operated on for a mulberry calculus, and went on tolerably well till the eleventh day, when he complained of pain in the left iliac region, greatly increased by pressure. In a day or two it extended to the groin—then the thigh—and ultimately the leg; bearing an exact resemblance to phlegmasia dolens of women. The swelling, pain, and stiffness continued for several weeks. The usual means were employed, viz. leeches, fomentations, liniments, &c.

A case of this kind has lately fallen under our notice, and was seen by Sir B. Brodie, Dr. Chambers, Dr. Johnson, and others. The patient was a medical gentleman, who fell into a bilious and ultimately a typhoid fever, which lasted a month, when an extensive slough formed on the sacrum, nearly laying that bone bare. When the slough had entirely separated, and when the extensive wound was filling up with healthy granulations, pain occurred in the left groin, and inflammation spread down the thigh, in the course of the great vessels, with as great tumefaction as ever occurs in puerperal phlegmasia. The swelling was pale—partly elastic—partly œdematous, and the pain very distressing. Leeches, fomentations, mercury, liniments, and all the usual means were employed; but the disease ran a long course, and its sequelæ have not yet disappeared. This poor gentleman had three narrow escapes from death:—first, the fever—secondly, the slough—and lastly, the phlegmasia dolens.

---

### Mr. R. Phillips, Jun. on a New Method of forming Iodide of Potassium.

Mr. Phillips having occasion to employ the Iodide of Potassium, procured six samples of it from different chemists, and found such impurities as evince the necessity for a good method of forming it. After pointing out the objections to the processes usually resorted to, Mr. Phillips goes on to say:—

"Another method is by boiling lime with iodine, thus forming an iodate of lime and an iodide of calcium, fusing the mixture to decompose the iodate, and then precipitating the lime by means of carbonate of potash, a carbonate of lime and an iodide of potassium being formed. In trying this process, it occurred to me that if I could add a body which would decompose the iodate of lime, the trouble and loss of fusion would be avoided, and this body I found in the protoxide of iron. It is easily prepared by precipitation with the alkalies from the protosulphate of iron. The precipitate must be washed by decantation, on account of its being rapidly converted into the peroxide, if exposed to the air until the solution gives no precipitate with chloride of barium. By this process the iodate was decomposed, its oxygen uniting with the protoxide of iron to form peroxide; it was, however, so mixed with carbonate of lime, as to be valueless. I found that on mixing the iodine directly with the carbonate of potash, the latter was converted into iodide of potassium and iodate of potash. The following is a diagram of the decomposition that ensues, except that potash is substituted for carbonate of potash, as the carbonic acid is expelled.

1 atom of Iodate of Potash.

1 atom of Iodic Acid. 1 Potash.

5 atoms of Potash: 5 Oxygen, 5 Potassium — Iodine 1, Iodine 5: 6 atoms of Iodine.

5 atoms of Iodide of Potassium.

"The protoxide of iron was then added, when the iodate of potash, in the same manner as the iodate of lime, was decomposed, with the following changes:—

12 atoms of Peroxide of Iron.

12 Oxygen, 12 Iron: 12 atoms of Protoxide of Iron.

1 atom of Iodate of Potash: 6 Oxygen, 1 Potassium, 1 Iodine.

1 Potassium, 1 Iodine: 1 atom of Iodide of Potassium.

"Peroxide of iron being a sesquioxide, that is to say, containing one atom and a half of oxygen combined with one of the metal, it is necessary, in order that the whole of the iodate be decomposed, that for every atom of carbonate of potash used, there should be two present of protoxide of iron. The quantities, therefore, to be taken, are two atoms or 280 parts of protosulphate of iron, which are to be decomposed into two atoms of protoxide, one atom or 125 parts of iodine, and one atom or 83.5 parts of carbonate of potash, P. L. On account, however, of the liability of the protoxide of iron to become peroxide, it is better to take rather more of the protosulphate.

"The protoxide, when washed, is to be mixed with the iodine and carbonate of potash, the proportion of water employed being half a pint to one ounce of iodine. The mixture is at first to be gradually heated, and then boiled for half an hour. The solution afterwards ought to be exactly neutral to slightly reddened litmus paper; if iodine be found in excess, which may be proved by the addition of a solution of starch to a little of it, more of the carbonate of potash must be added; if, on the contrary, it be alkaline, more of protoxide of iron and iodine.

"The sesquioxide of iron remaining after being washed until the liquor gives, when acidulated with nitric acid, no blue colour if solution of starch be added, may be heated red hot for a few minutes, a beautifully pure preparation remains, which, on account of its easy solubility in hydrochloric acid, might be used with much advantage in the preparation of the tincture of the sesqui-chloride of iron. I have several times mentioned a solution of starch: it may be as well to observe, that it requires a temperature of about 170° to dissolve this substance."—*Pharmaceutical Journal,* August 1844.

---

Gonorrhœal Orchitis and its Treatment by Narcotics. By J. Gay, Esq. F.R.C.S.E. Surgeon to the Royal Free Hospital, &c.

Cases are recorded in some of the recent numbers of the Lancet shewing the efficacy of narcotics in that, the most common, form of orchitis which is met with after the fourth week of gonorrhœal urethritis; and pages 602, 3, and 4, of the same Journal contain an article in which Mr. Gay's views of the nature of this affection, and the reasons which led him to adopt this mode of treatment are set forth, of which the following is a summary.

The prevailing symptoms of this affection are:—"general debility shown by unusual pallor of the face and mucous membranes: the skin covered with large drops of perspiration on slight exertion; quick pulse; furred tongue; high-coloured urine, and constipation. The local symptoms set in with dull aching pain about the testicle and epidydimis, increased on pressure; and the formation of a dense tumor, generally in the most depending portion of the epidydimis, and circumscribed by its investing tunic. Inflammatory action commences in the part, and extends itself to the tissue of the testis; the whole swells, becomes more and more dense, and so acutely painful that the slightest touch can scarcely be borne. The vas deferens, cord, and scrotum, next become involved in one swollen, painful, mass, the veins of the latter tunic becoming exceedingly congested with dark blood. As the inflammatory action proceeds, so does the condensation of the tissues (first observed as a small hard tumor of the globus minor) extend upwards, involving in its progress the lowest portions of the testis and epidydimis, until, eventually, in extreme cases, the testis and epidydimis become one dense compact mass."

These symptoms are introduced by a dull pain in the testicle, only discovered by pressing that organ: and hence it is of the utmost importance during the continuance of that stage of a gonorrhœa favourable to these affections, that the testis should be constantly watched, so that any increased susceptibility of touch may be detected; and by the use of appropriate remedies at this period, the more serious consequences be averted. The spread of the inflammatory action from the testis to the adjacent and tegumentary tissues in all probability depends upon the obstruction which the abdominal ring offers to the free return of venous blood consequent upon a swollen condition of the cord from effusion into its cellular tissue; and it may admit of some doubt whether the parietes of the inguinal canal may not, under these circumstances, *spasmodically* contract upon the swollen cord; for it is a remarkable fact, that upon the administration of a remedy in such cases, whose tendency is to allay spasm, the inflammatory action of the cord and integuments which is last set up is most frequently the first to subside.

The following case will illustrate this pathological view. In December, a man named S—, ætat. 48, was brought into the Royal Free Hospital, having fallen from a scaffolding, and was so injured that he died. Two days before his death, and three days subsequent to the accident, a *violent* attack of orchitis came on, the inflammation involving, in order, the epidydimis, testes, cord, and scrotum. The parts were all very much swollen as high as the external ring. He had discharge from the urethra. The veins over the scrotum, and throughout the inflamed parts, were excessively distended; the testes and epidydimis, on being cut through, showed the first stage of inflammation, the cut surfaces exhibiting a roseate tint, and a vast number of small vascular points throughout. The globus minor of the epidydimis, and most depending portion of the testis, were, by a shade, of a deeper red than the other parts, and slightly more dense; the structure in these parts being rather more distinct, in all probability, from the commencement of effusion of lymph between the tubuli. There was a little fluid, probably not more than in health, in the tunica vaginalis. The sheath and tisues of the cord were excessively distended by serum and venous blood.

Amongst the causes of this affection, the chronic disease of the urethra which maintains the discharge, is obviously the most important: but it does not stand alone, for such diseased action may continue for months and yet no affection of the testis supervene. Combined with it there must be other agents and these are ever of a depressing character, and calculated to bring about those general indications of debility which the symptoms that have been recounted present; such as the long use of purgative medicines, low diet, mental disquietude, the disuse of accustomed invigorating exercises, &c. The immediate cause will generally be found to be either over-exercise, especially walking, a blow, *constipation*, improper stimuli applied to the urethra, sometimes the operation of a drastic purgative, or several of these combined.

The orchitic affection *commencing* in the testis, and thus showing its purely sympathetic character, differs essentially from that form to which M. Ricord refers, in which it occurs "from succession or continuity of tissue, or *by extension of the inflammation from the urethra to the canalis ejaculatorius and thence to the vesicula seminalis, canalis deferens, and lastly to the epidydimis,* as demonstrated by pathological anatomy." This form is rarely to be met with, occurs in the acute stage of gonorrhœal urethritis, and is amenable only to active antiphlogistic treatment.

With regard to the influence of the discharge from the urethra upon the orchitis, Mr. Gay does not regard it as a "translation of the inflammation," as Mr. Hunter supposed. Mr. H. says, "a swelling in the testicle coming on shall remove the pain in making water and suspend the discharge, which shall not return till the swelling of the testicle begins to subside; or, the irritation in the urethra first ceasing, shall produce a swelling of the testicle which shall continue till the pain and discharge return, thus rendering it doubtful which is the cause and which the effect. I have, nevertheless, known cases where the testicle has swelled, and yet the discharge has become more violent; nay, I have seen instances where a swelling has come on after the discharge has returned with violence, and remained as long as the swelling of the testicle." The explanation of these admirably described and apparently anomalous facts, Mr. Gay conceives to be as follows. Orchitis setting up during the continuance of a discharge from the urethra, acts upon the primitive seat of disease as a counter-irritant, and may or may not be effective in arresting the discharge; the failure to do so being for the same reasons (whatever they may be) that the application of blisters with the view of removing chronic disease in any organ does not in all instances effect its purpose. But there can be no doubt that, as the chronic disease of the urethra was the cause, without which the subsequent orchitis could not have existed, the speedy removal of the one is essential to the cure of the other. Hence the popular plan of renewing the discharge from the urethra by means of bougies and other irritants is most unsafe and unphilosophical; on the other hand, the employment of a mild injection of sulphate of zinc, or di-acetate of lead, or, should the disease have existed long, of a strong solution of nitrate of silver, will be found as safe and necessary as it is reasonable.

From these data Mr. Gay infers:—

"1. That by a protracted gonorrhœal discharge from the urethra, and the remedies used for its cure, the general health suffers, and a state of diminished tonicity, with irritability of the whole system, is produced.

"2. That from sympathy with the seat of discharge the nerves of the testis and epidydimis become affected with an especial degree of morbid sensibility. This *particular* sympathy of the secreting apparatus with the urethra is constantly noticeable. An injection, which gives pain to the urethra, will always impart uneasiness to the testicle.

"3. *That thus prepared,* local exciting causes will produce inflammation of a low order in the cellular tissue, connecting the seminal tubules (for it is obviously that element of the gland that the diseased action, in all its stages, occupies, from the fact that there is no obstruction to the passage of the seminal fluid during an attack of orchitis).

"4. That the results of this action are effusion and venous congestion, from a want of power in the veins (owing to their participating in the systemic asthenia), to return the increased supply of blood to the testis.

"5. That congestive inflammation of the *adjacent and tegumentary textures* with its consequences will follow; and probably an extreme of this condition will be produced by the obstruction to the free return of venous blood from the testis, which the external abdominal ring has been found to offer."

Hence Mr. Gay deduces the practical fact, that opium, hyosciamus or other narcotic, so given as to produce indubitable evidence of general narcotism, does and will speedily effect a cure of this special form of orchitis by allaying that general and local irritability which is presumed to exist as the immediate cause. But it is found imperatively important that the bowels should *first* be thoroughly evacuated by a brisk and cleansing purgative; and the aperient should be repeated in three or four days if the bowels tend to constipation, which is a constant feature in this affection. The anodyne Mr. G. uses is the tincture of hyosciamus, of which from 15 drops to a dram may be given every four or six hours, according to the urgency of the case, and at the same time cold applications should be made to the scrotum, and the patient be required to remain on his back with his hips raised. This treatment is however but another plan of fulfilling the same intention for which Frieke's system of bandaging was devised, but it is more extensively applicable, more easily employed, and equally remedial.

---

### Lepra Græcorum Contagiosa ?

### Nova Pestis Adest!

The inhabitants of New Brunswick are at this moment labouring under a Lepra-Phobia, little inferior in violence to the Cholera-Phobia which prevailed in this country, during the eventful years of 1831-2! The Government, like the Parent Government, issued a Commission to investigate the terrible malady; and the following is a critique on the "Report" of the said Commission.

"*Brief Remarks on 'The Report' laid before the Government of New-Brunswick by the Medical Commission appointed by the Lieutenant-Governor to investigate the Nature, Causes, &c. of a Disease termed Leprosy, prevailing in certain French Settlements in that Province, bordering on the Gulf of St. Lawrence.*— By Alexander Boyle, M.D.

"A representation having been made to the Lieutenant-Governor of New Brunswick, that a disease of a highly malignant and loathsome nature, receiving the name of *Leprosy*, had been discovered among the inhabitants of certain French Settlements in the Counties of Gloucester and Northumberland, in that Province; a Commission, composed of four Medical Gentlemen and the French Clergyman residing in that district, was appointed for the purpose of investigating its nature and causes, and suggesting such measures as might be judged necessary for its prevention.—The Commission entered on this enquiry in March last, and the result is now before the public; and a sum of £1000. has been granted by the Provincial Legislature, to enable the Government to carry into effect the measures proposed.

"As they have 'unanimously recommended the erection of a Lazaretto, the removal of the sick, and their strict seclusion in that establishment,' as means necessary to guard against the extension of this malady, which, they consider as not only hereditary, but *contagious*; I have been induced to examine some of the more prominent facts which they have brought forward in support of this opinion; with the view of determining how far it is expedient, on this occasion, and on the grounds exhibited, to have recourse to measures involving extreme hardship on individuals, and not devoid of injury to society, and which ought never to be adopted under the sanction of causes which admit of doubt;—and with what probability of success those measures are calculated to accomplish the end proposed.

"It may, in the first place, be observed, that much confusion has been introduced into medical writings, in consequence of doubts respecting the disease to which the name of *Leprosy* more properly belongs; arising, as is supposed, from the inaccuracies of the translations of the Greek and Arabian authors who treat of this subject, and who have sometimes described, under the same title, three different affections, very distinct in character, namely, the Hebrew Leprosy, a scaley disease; the Elephant, or Barbadoes leg; and the 'Tubercular' disease, now under consideration. It is, however, at present unnecessary to enter farther upon this subject, as the disease in question must be viewed under the aspect presented to us by the cases given in the 'Report,' where it is classed under this last denomination; and, as observations of *modern* date have removed much of the perplexity by which it was obscured, there is less difficulty in assigning to each its proper place in nosological nomenclature.

"The facts collected, and which form the subject of the official document, are interesting; and the cases are detailed with a degree of accuracy and clearness, leaving no doubt as to the existence of a disease which has all the characteristic symptoms of the 'Tubercular Elephantiasis' of modern nosologists, the 'Juzam' of the Arabians, and the 'Lepra Græcorum' of the middle ages, by which its identity is fully established, and which, therefore, need not now be repeated.

"It is stated in the 'Report,' that 'no *positive* conclusion could be drawn as to its original appearance in this quarter;' but, that according to the information that was received from the oldest settlers, the first case occurred in the person of Ursule Laudre, about the year 1817, nineteen years after her marriage with Joseph Benoit, of Tracadie, a small French settlement situated in a part of this Province, bordering on the Gulf of Saint Lawrence, and where she went to reside after her marriage. Her father, mother, and their children, *nineteen* in number, appear, with the exception of Ursule alone, to have been perfectly healthy. She was married about the year 1798 or 1799, and having been affected ten years *before* her husband, (for, it appears, he also became affected with it,) it would be absurd to suppose that she possibly could have received the infection from *him*; neither can we suppose that he could have contracted it from her, for he continued free from it until three years before her death, which happened in 1829; a period of twenty-six years after their marriage, and of his having lived with her about ten years *after* she had shewn all its unequivocal symptoms. After the birth of her fifth child, Ursule ceased to bear children: and it is stated that, from that time, her health continued to decline for six or eight years, when the disease was no longer doubtful.

"Upon the whole, other facts stated in the 'Report' are not more favourable to the existence of *contagion* as a cause of this disease, than those already mentioned; for it is not shewn from whom Ursule received the infection, nor is it proved that she communicated the disease to any one, either by direct contact, or through the medium of substances imbued with a contagious principle. There is, indeed, no sort of analogy, as far as those facts go, between this disease and those of a contagious nature; in most of which, we can, almost, as it were, detect the contagion in its passage; and it is only by analogy we can reason on the subject. The latent period in contagious diseases is never long—

being only two or three days, sometimes less, and seldom exceeding three weeks; whereas, in the present instance, several years of continued intercourse intervened from the first communication with the infected person to the time of its appearance in the individuals exposed.

"As it is admitted by all that 'Tubercular Elephantiasis,' or the Greek Leprosy, as it is sometimes called, is an *hereditary* disease, and like Scrofula may pass one generation and appear in the next, when concurring causes favour its development; the taint in one of the nineteen children of Laudre *pere* (Ursule) may have been derived from a source to which, at this distance of time, it cannot, perhaps be traced; and the case of the only *one* of her five children who was affected, must be referred to the same category; while the exemption of the other four, though living in continued intercourse, completely overturns the doctrine of *contagion*, and is in perfect analogy with the facts observed in Scrofula and other diseases which are transmitted from parents to their posterity, affecting some and sparing others, according to circumstances not often very evident or easily explained. The case of Frances Savoy is not less decisive. She has a family of six children, and only *one*, a boy of eight years of age, is affected. She herself has had the disease four years and a half, in a severe form; 'her husband sleeps with her every night, and is in perfect health.' P. Savoy's case is a severe one. He has a wife and four children, but all of them are apparently healthy. The case of T. Robicheau bears equally on the same point. He is about fifteen years of age, and has been affected six years; and his uncle was recently carried off by it; marking, decidedly, *hereditary* transmission in that family.

"It must, indeed, attract particular notice, as a singular feature in the 'Report,' that, as regards *contagion*, so many of the facts related militate strongly against the conclusion which has been deduced from them, on this important question.

"The affected district is situated between the Bay of Chaleur and Miramichi River, and the intercourse between it and Canada, Prince Edward Island, Nova Scotia, and the adjoining Counties of New Brunswick, has been frequent and uninterrupted; and, though the disease was known to have occasionally existed in some parts along the Gulf shore at a date anterior to that assigned to it in the 'Report,' it had not, it would appear, been propagated to any of the above-named places, nor been considered of that importance, or so frequent at the time, as to attract particular notice, or excite any serious alarm; and it is only now, though twelve cases terminated fatally, and, as appears by the 'Report,' after it had prevailed in Tracadie for a period of twenty-seven years, that the attention of the Legislature has been called to it, as fraught with public danger, and requiring rigid measures of prevention!

"That the original source of the disease is to be detected in any geological peculiarity observable in the tract of country above-mentioned, is not very probable; atmospheric causes are temporary, though general in their operation, giving to diseases an epidemic character; and the climate and cultivation of the soil, if not much improved, certainly are not deteriorated. The habits and mode of living of the French inhabitants in that part of the Province of New-Brunswick, it may also be remarked, have always been pretty much the same since its first settlement, with those in other parts of British North America, except, that the fish, which constitutes almost their sole article of food, is not, perhaps of the same wholesome quality as among some of their neighbours, owing to their poverty, which often prevents them from procuring the means necessary to preserve it for use. Some further facts, therefore, are wanting to render our conclusions as to the primary cause of its appearance in that locality satisfactory; and it is to be regretted that it could not now be traced further back than the case of Ursule Laudre, in 1817.

"The inhabitants of this district are of the Norman race, and descended from those Frenchmen who visited North America at a very early period of its settlement; and by whom, it can scarcely be doubted, the disease was brought to this part of the world; and the superb structure erected by Henry II., Duke of Normandy, at Caen, in 1160, for the reception of Lepers, but long since appropriated to another use,) attests to this day the prevalence of the disease, in former times, in that country, where it had been, in a manner, naturalized since the time of the Crusades. It is of no small importance, therefore, as tending to throw a ray of light on this part of the inquiry, to know that Ursule's father was an 'Acadian,' that her mother was of 'Caranuette' in the Bay of Chaleur, and her husband from 'Tracadie,' on the Gulph Shore; for it is also known that the *Baie de Chaleur* and the north-east shores of *Acadie* were places where those adventurous navigators first encountered perils; and the disease may have even disappeared for a season, and at different periods from that early date sprung up again among their posterity, who appear still to inherit the original taint.

"From the facts stated, it does not appear what has been the total number of cases from the year 1817 until the present time, although nineteen '*confirmed*' cases were found at the time of their visit; a proportion not exceeding, perhaps, that of any other disease, acute or chronic, which has appeared in this district during an equal period, reckoning from the average duration of the cases recorded in the Report; and it might, perhaps, be also ascertained that the deaths from any one of them were not less numerous than those from Leprosy, which, as far as we are informed, amount to twelve.

"Having thus taken a brief review of the principal data furnished by the Report, and on which the Commissioners found their opinion as to the *contagious* character of the disease, which alone could warrant recourse to the seclusion of the affected and the adoption of Lazaretto regulations and restrictions; it may not be deemed unprofitable or uninteresting, in a question which so deeply involves the comfort and safety of a large portion of society, to quote some *modern* authorities of the highest weight in the medical profession in support of the opposite doctrine, or *non*-contagious nature of this loathsome disease, which is universally acknowledged to be *incurable*.

"In an article in the 'Dictionair de Medicine et Chirurgie pratiques,' by Bouillaud, vol. 5, p. 425, he says, 'La contagion a été admise par certains auteurs dans une foule des cas ou elle n'existe realment pas. Quel est le medecin observateur qui ajoute foi maintenant à tout ce qui a été debité, par exemple, sur la contagion de la Lepre,' &c. And again, vol. 5, p. 435, when speaking of isolating the sick and of sanatory cordons, he expresses himself thus: 'Les attaques dont cette mesure sanitaire a ètè l'objet dans ces derniers temps, paraissent bien fondéas, et le moment n'est peut-etre pas eloigne, ou elle sera completement abandonneè.' To this effect, also, in an article in the same work, vol. 14, p. 19, by Ch. Londe, when speaking of the instructions relating to Quarantine, he says, 'Le Lepre arrive, dans *les Instructions,* en quartieme ligne; mais parmi les gens qui ont bien observe cette maladie, aucun aujourdhui ne croit plus a sa prorieté contagieuse; les dispositions atroces, prises contre les lepreux, sont tombees en desuetude, et ces malades sont admis parmi les autres dans nos hôpitaux, sans que jamais on se soit aperçu de la transmission de leur affection.'

"In a paper in the Cyclopedia of Practical Medicine, on 'Contagion,' by Dr. Joseph Brown, we find the following remark:—'There are two other diseases which were *formerly* considered contagious, Lepra Græcorum and Lepra Arabum, (Elephantiasis). The former is unquestionably not contagious; and from the observations made by the late Dr. Adams in the Lazaar-house at Funchal, there is every reason to think the latter equally devoid of that quality.'

"Numerous authorities might be quoted to the same purpose, but the above are conclusive; and the proofs of *hereditary* transmission are not less clear and cogent.*

* Rayer, Bateman, Alibert, Robinson, T. Heberden, Ainsley.

"Various external causes have been mentioned as producing this disease; and there is a general assent as to the agency of bad food, especially putrid fish, and vicinity to the sea shore. There is, however, in many instances, the greatest difficulty and uncertainty in accounting for its appearance; and the above, like those of a moral nature, of which a remarkable instance is recorded by the celebrated Alibert as coming under his own immediate care, (Precis, &c. vol. 2, p. 84) it is presumed, act chiefly by awakening a latent hereditary predisposition; and it must be confessed, some cases baffle all conjecture. The testimony of Sonini and Pallas is referred to by the same author as opposed to the doctrine of *contagion.*

"Having myself seen, some years ago, a well-marked case of this disease which leads to conclusions in perfect accordance with the opinions of those eminent men, I may be permitted, in this place, to bring it under notice. It occurred in a person about thirty years of age, a native of Antigua. He had laboured under it for a considerable length of time, and came to Nova Scotia for the benefit of his health, where he married an interesting young lady, about two years after I saw him. After this, he came to Saint John, and was under the care of Dr. Boyd and myself for about two months. His breath was extremely offensive, and his hands, face and legs were covered with blotches and tubercles of a livid, brownish colour; and some of them were in a state of ulceration. He afterwards went to New-Orleans, in a worse state than when he first left the West Indies; and died of this disease not long after his arrival. During the whole period of his illness, from the time of his marriage, his wife was most assiduous in her attentions to him, and occupied the same bed; but neither she nor any of the inmates of the boarding-house where they lodged for nearly twelve months, and with whom they had daily intercourse, ever showed the least mark of the disease. He had no children; and after his death, his wife returned to Nova Scotia, where she has continued to enjoy perfect health.

"In the course of the above remarks, I have purposely abstained from all opinions respecting the operation of contagious miasmata, or the manner of their introduction into the blood, as foreign to the object of the present enquiry; and as chemistry has, hitherto, thrown but feeble light on the nature of their elementary constituents, or the changes they effect on the circulating fluids.

"Several important organs undergo a morbid alteration of structure during the progress of this disease; but, as its pathology is unconnected with the question at issue, it need not now engage our attention.

"It is generally admitted that Pulmonary Consumption is, according to the common acceptation of the term, an hereditary disease, usually developed by the action of exciting causes. In some countries, it is also deemed contagious; and the houses which have been occupied by persons who die of this complaint, are always left deserted. In New-Brunswick, its occurrence is very frequent; and, though not deemed contagious, it may certainly be pronounced *incurable.* So far, the parallel between it and Leprosy is strictly correct; but surely, it would not be recommended that those persons labouring under Pulmonary Consumption throughout the Province should be torn from their families and left to die in a Lazaretto; nor can it be imagined that any such measure would be proposed to prevent the continuance of so great a scourge of the human race; as it is sufficiently evident, how fruitless the attempt must be, by the removal of a few, to arrest the progress of a disease whose germ, unhappily, is but too widely spread among mankind, and whose extinction is beyond the reach of legislative power.

"During the prevalence of the Asiatic Cholera in Europe erroneous opinions regarding its contagious character led to the adoption of similar measures of prevention; but experience has since taught us how useless and unnecessary they were, and how injurious to the interests of society; and the novelty of the disease among us, and its still inexplicable nature, afford the only vindication of the course pursued during that period of general consternation. In no part of Europe was Leprosy so prevalent at one time as in France, from the eleventh to the sixteenth century. It is now chiefly confined to the tropical and equatorial regions, being rarely met with in Europe since the seventeenth century; and the advanced and more refined state of society accounts for its gradual disappearance; while greater experience has banished all dread of its supposed contagion, and opened their noble hospitals to the admission of Lepers, without distinction, among other patients; and numerous Lazarettos of great extent have long been converted into retreats for the aged and infirm, or like purposes of general philanthropy and benevolence.

"I trust it will appear, from what I have said, and the authorities I have referred to, that any measures of prevention which require seclusion of the sick, much less the severity of Lazaretto regulations, are not warranted, on this occasion, by the facts on which their adoption is grounded; and as their uselessness and impolicy have nearly expunged them from the code of civilized nations.

"The formation of any hospital *exclusively* for the reception of leprous patients is also objectionable, as entailing an endless expense on the Province; especially if County Hospitals already exist, or similar establishments, into which, as experience has abundantly shewn, they may be received without the remotest injury to other patients; and of which, the 'Report' itself furnishes ample proof. The disease being incurable, little good is to be expected from hospital treatment; which, at best, could only be palliative. Much, however, may be done to obviate some of the causes which render the human body liable to various diseases arising from noxious emanations, as well as from atmospheric influence and hereditary predisposition, by inculcating the necessity of a proper hygiene throughout the French population of that district; by which the constitution may be enabled to resist the influence of external agents; and without which, it is to be feared, the disease will finally cease only with the extinction of the race.

"With this view, enquiry should be made by the Clergymen of the district to which the disease is confined, into the habits and means of subsistence of the several families under their pastoral care,—their occupations, their clothing, their ordinary food, and the state of their habitations; and according to their age, sex and number of children, such pecuniary aid should be given as would relieve their wants, stimulate their industry, and, not less important, dispel despondency and grief, by shewing them that a general interest had been awakened in their favour, and that the present evil was not altogether beyond the reach of relief. The appropriations of money from the public purse for this humane and benevolent purpose should, for obvious reasons, be entrusted to the hands of the Roman Catholic Bishop of New Brunswick and the Pastors of the district; to be distributed at their discretion and responsibility, rendering an account of the disbursements to the Executive Government of the Province, which alone should have power to controul them."

*St. John, (New-Brunswick,) May* 15, 1844."

---

HYSTERIC SPASM OF THE DIAPHRAGM. By JOHN RINGLAND, M.D. (Dublin Medical Journal).

The following are the results of the observations of several cases of this affection which Dr. Ringland has lately met with. That the diaphragm should be affected to a considerable extent in cases of hysteria, he remarks, need not be wondered at, when we consider that no organs sympathize more with the nervous system than those engaged in the function of respiration; but in these cases the whole of the respiratory apparatus was implicated to a degree almost incompatible with existence.

The attack commenced at night, without any premonitory symptoms whatever, with the sensation of suffocation, as though a cord was tightly constringing the throat; there was much distress experienced in attempting to swallow even the smallest quantity of saliva. There was most intense pain in the region of the stomach, passing backwards from the scrobiculus cordis towards the spine, then taking the direction of the lumbar portion of that column, it terminated in the lower part of the sacrum.

This pain was most intense in the situation of the diaphragm, and became less so as it progressed towards the sacrum. It was described by the patients themselves as though the part affected was torn with hot pincers. There was considerable pain experienced on making pressure over the lumbar vertebræ. The breathing was much laboured, requiring the patients to remain in the sitting posture, the distressing sense of suffocation rendering it impossible for them to remain in the recumbent position. The respirations were upwards of sixty in the minute; the expirations were much longer, and accompanied with less pain than the inspirations; during the latter, the ribs and clavicles were elevated to an extraordinary degree. A most distressing hiccough recurring in fits at short intervals, left the patients at times almost breathless. There was slight fulness of the abdomen, with tympanitis, but no pain on pressure, excepting when it was made upwards towards the diaphragm, and then the other symptoms became, if possible, slightly aggravated. The skin was quite natural, but the extremities were very cold. Urine was voided in larger quantity, and paler in colour than usual. Bowels always regular. Pulse not altered in any way from the healthy condition.

The administration of anodynes in combination with some of the volatile spirits (as ether, ammonia, ammoniated tincture of valerian), together with warm applications to the feet and abdomen, afforded temporary relief, but the attack recurred again in a few hours without any abatement of the symptoms. The same plan of treatment had a like effect, till at length, on the approach of morning, a deep sleep came on which lasted for several hours, and the patients awoke, free from all cause of complaint.

When the patient did not submit to a continuous treatment for several days, the attack again returned, and the same remedies had to be resumed, and with a like success. However, a permanent cure was effected by the administration of the fœtid gums, combined with the volatile spirits; anodynes on the slightest threatening of a recurrence of the attack; sinapisms to the sternum and spine; and plasters of belladonna to the loins. These measures, when steadily adhered to for seven or eight days, permanently cured the attack.

No class or condition of persons, nor habit of body, seemed exempt from its attack. Of those Dr. Ringland attended, one was a lately married female; another, a lady who was nursing her second child; another was at that period of life when the catamenia are about to present themselves; and, in the last, the menstrual function had been fully established.

Again, one of the four was in the higher rank of life; one had been reduced to considerable poverty, but was, at the period of the attack, in comfortable circumstances; while the other two belonged to the better portion of the lower rank of society. Besides this, three of the patients were extremely fat, and of middle size, whilst the fourth was tall and slight. Five cases are related at full length.

---

### Liquid Medicines. (Medical Examiner).

It has been found that fifteen grains of sulphate of quinine exhibited in infusion of senna, are more efficacious, as a *tonic*, notwithstanding the aperient quality of the vehicle, than twenty-four grains of quinine taken in pills. M. Panezza accounts for this difference by supposing that the senna, by augmenting the peristaltic action of the alimentary tube, and increasing the secretions of the bowels, excites the production of a fluid well adapted for perfectly dissolving the quinine, and in that state to be applied to a much greater surface of absorption than if it passed along the canal in the form of pills. There may be something in this reasoning. Indeed we never had much confidence in quinine pills, or even steel or other tonics in that shape.

---

### PROPHYLAXIS PTYALISMI.

All attempts at curing mercurial ptyalism by medicine, being now given up by universal consent, the idea of prophylaxis has struck Professor Schoeph, of PERTH. We suppose the worthy Doctor is Professor of "DENTISTRY," for the preventive check is a tooth-powder composed of alum and cinchona! We can inform Dr. S. that a far more certain Prophylaxis Ptyalismi is, the non-administration of mercury.

---

### OBSERVATIONS ON THE TREATMENT OF ACUTE RHEUMATISM BY CINCHONA BARK. By JOHN POPHAM, M.B. (Dublin Medical Journal, September).

Though far from agreeing with Haygarth and his followers in their excessive approbation of this remedy, Dr. Popham considers it as one of too great value to be overlooked by the profession. At the commencement of the complaint, and while the symptoms were severe, the administration of the bark was attended with no good effect, but the results were different when it came in at the turn of the disease. In twelve cases of acute rheumatism in which it was given at a period varying from the fifth to the tenth day after the attack, nine were perfectly cured in less than three weeks, without relapse or loss of strength, and without those unruly pains and aches that so often survive the original attack.

The cases in which it was most successfully employed were those of fibrous rheumatism or rheumatic fever properly so called. When it appeared at all probable that either the pericardium or heart was affected, the bark was not exhibited, at least until the inflammatory symptoms were checked. In capsular rheumatism the bark seemed to disagree with the acute stages, aggravating the symptoms, but in very chronic cases it seemed of service.

The conclusions at which Dr. Popham appears inclined to arrive, with regard to the administration of this medicine, are these :

"That it is important to procure due evacuations previous to the exhibition of the bark, except the patient be greatly deteriorated by constitutional debility, or the protraction of the disease.

"That it is more *quickly* successful when the disease is *early* combated by depleting measures, than when inefficiently managed at the onset, and allowed to take root in the system.

"Hence that is more likely to extinguish the disease and prevent chronic infirmity in the sequel of *first* attacks being uncomplicated, than when a habit has been formed by reason of repeated relapses.

"That the periodicity of the symptoms either peculiar to the attack, or produced by treatment, and the duration and apyrexia of the intervals, afford strong presumptive arguments for the use of bark.

"That bark is especially called for in cases where there is complete atony of the cutaneous vessels, so that the skin is unceasingly pouring out acid colliquative sweats, giving it a dull and parboiled appearance, at the same time that the pains are abated, and the pulse small and indicating debility.

"That to produce its effects, quantity is not by any means so essential as in intermittent, and that large quantities, especially of the sulphate of quinine, derange the stomach in many cases, and bring back the fever.

"That it is judicious to administer it at the periods of remission, and stop it at the return of the exacerbations.

"That it is injurious when important visceral disease co-exists, and is especially contra-indicated in cerebral or the acute stage of cardiac complications.

"Lastly, that in the synovial variety, it is inferior to other modes of treatment, but in persons of a rheumatic diathesis, when, from the long continuance of the disease the strength has suffered, and disfiguration of the joints has occurred without serious destruction, a course of bark, combined with sulphur, &c., often prevents the recurrence of subacute attacks, and promotes the absorption of the effused synovia."

---

Dr. Hamilton on the Jamaica Dogwood. (Pharmaceutical Journal, Aug. 1, 1844.)

Dr. Hamilton, during a visit to the Antilles, was struck by the powerful narcotic effects on fish produced by the bark of the roots of the *Piscidia erythrina*, or *Jamaica dogwood.* Thinking that this might be of utility as a medicine, he found that the tincture, prepared by macerating the bark of the roots gathered during the period of inflorescence, and before the appearance of the leaves (the piscidia being one of the few deciduous trees indigenous within the tropics), in four times its weight by measure of *rectified* spirit for twenty-four hours, and filtering, was the only eligible mode of preparation. The following was his experiment, and its result.

"Having been for some time a martyr to the tooth-ache, which had deprived me of my natural rest, I determined to make the first trial of my new medicine upon myself; and accordingly on going to bed, mixed a drachm-measure of the tincture with a rummer of cold water, and drank it off—waiting to observe its effects. Soon after receiving it into the stomach, I experienced a violent sensation of heat, which gradually increased in intensity, awakening in my mind a suspicion that the predictions of my friends, who assured me I should poison myself, were on the eve of fulfilment. However, the deed was done, and I resolved to abide the issue without flinching. The sensation of burning gradually extended itself to the surface, and while I was considering what antidote I ought to employ, a profuse diaphoresis burst out from every pore, and a sleep the most profound I ever experienced arrested me so abruptly, that I remained motionless for the whole night, with the uncorked phial in one hand and the glass out of which I had taken the dose in the other, till after the sun was high above the horizon on the following morning, a space of twelve good hours, when I first returned to consciousness, free from every pain or ache, and without any of those unpleasant sensations which invariably succeed to an overdose of opium.

"I had certainly taken a larger dose than was, perhaps, necessary; but the result was most triumphant. I afterwards employed it with equal success, as a topical application, in a number of cases of carious teeth, introducing it on a dossil of cotton into the diseased cavity; and after a single application, I never heard of a return of pain in that tooth. Wishing to compare its powers with those of opium, I took equal measures of water, containing the animalculæ of the mosquito, and having dropped into one glass as much of the tincture of opium as was necessary to make them fall motionless to the bottom, I added an equal number of drops of the dogwood tincture to the other, with a corresponding effect. I then decanted the supernatant liquor, and washed the mass of animalculæ in each glass with fresh filtered water from the dripstone; after a few ablutions, those which had been stupified by the tincture of opium recovered, and swam about with their wonted vivacity, while all my efforts to revive those acted upon by the dogwood proved ineffectual."

Dr. Hamilton subjoins certain cautions as absolutely necessary to be kept in view. "First, the bark of the roots should be collected about the period of the full moon, in April, at which time the tree is in full flower, or coming into flower, and the leaves have not yet unfolded. Next, that the best rectified spirit alone should be used in making the tincture—the active principle of the bark being only soluble in spirit, and precipitating on the addition of water, with which it makes a milky compound. It is possible that a longer maceration than I employed might be successful in extracting more completely the active principle; but it might be desirable to divide the tincture so obtained, keeping that made during the first twenty-four hours distinct from the second, or making a portion with twenty-four hours' maceration, and another portion with forty-eight hours' or more; and comparing, by some common test, the relative qualities of each. The following is the formula for the tincture I used:

℞ Pulveris crassi Corticis Radicis Piscidiæ Erythrinæ, ℥j.

Spiritus Vini Rectificati, fl℥iv.

Macera simul per horas viginti et quatuor in vasi aperto et cola. Dosis fl℥j. et infra ex haustu aquæ puræ."

---

SIMPLE METHOD OF PREPARING THE PILULA FERRI IODIDI. (Pharmaceutical Journal, August, 1841.)

Take of iodine 127 grains, iron wire, about the thickness of a thin quill, half-an-ounce, distilled water 75 minims. Agitate them briskly together in a strong ounce-phial, provided with a well-fitted glass stopper, until the froth which forms becomes white, which will happen in less than ten minutes. Pour the liquid upon two drachms of finely-powdered loaf-sugar in a little mortar, and triturate immediately and briskly for a few minutes; add gradually a mixture of the following powders, viz,, liquorice powder half-an ounce, powder of gum Arabic a drachm and a half, and flour one drachm. Divide the mass into 144 pills.

Each pill contains about a grain of iodide of iron.

In operations on the large scale, the bottle ought to be wrapped in a strong towel, in case of an explosion being caused by the evolution of steam from the heat produced; and even on the small scale, the stopper must be held firmly, otherwise it will probably be blown out and the materials lost.

---

THE RESPECTIVE RIGHTS OF CHEMISTS AND APOTHECARIES.

Every-body knows the jealousy felt by chemists and apothecaries, on the subject of their respective rights, privileges, and encroachments. The apothecary accuses the chemist of prescribing—the chemist replies that the apothecary retails medicines. There are some observations in the Pharmaceutical Journal, of a very moderate character, which, perhaps, may be read with advantage by both parties. We must say, however, in anticipation, that the apothecary has a better *right* to retail medicines, than the chemist has to prescribe them.

"It is probable that some of our brethren, who are desirous of invading the province of medical practitioners, may cordially approve of a measure which leaves them at liberty to use their own discretion, and even to visit patients, without becoming liable to any kind of interference. But those who take this view of the subject will do well to consider, whether this unlimited licence might not be attended with some disadvantage. If the members of our body are to be allowed to encroach, *ad libitum*, on the medical department, is it likely that we can obtain any protection against the grocers, oilmen, and hucksters, who may, in their turn, feel disposed to embark in a ruinous competition in the sale of medicines? May we not also expect, that if all restraint upon quackery is to be at an end, 'doctors' shops' will rise up like mushrooms against us; and that what we lose on one side will much more than counterbalance what we gain on the other?

"While we claim a certain amount of liberty, as vendors of medicines, in giving our customers the requisite information respecting the properties and doses of the remedies, it may reasonably be questioned, whether we should be gainers by overstepping the boundary, and professing to act in every respect as medical men."

"But while the Apothecary sells medicine not only to his patients, but to any retail customers who present themselves, the Druggist in self-defence is obliged to overstep the bounds of his office, knowing that if he were in every trivial case to refer his customers to the Apothecary for advice, he would by so doing lose the sale of the medicine altogether. But if it were the custom for each party to perform the duty for which he is by his position and education best qualified, the patient would be better served, the prescriber and dispenser would be more adequately remunerated, and the jealousy between them would cease."

We fear that such cordial agreement is improbable. The tendency of things appears to be, that the general practitioner should grow more of a chemist—the chemist more of a general practitioner. Most young apothecaries now commence with an open shop, and as much retail custom as they can procure. As they get on, the open shop is replaced by a private one. Whichever party began the war it is now waged actively, and is not likely to come to an end in a hurry. Should Sir James Graham's Bill pass, matters, we fancy, will grow worse.

---

PREPARATION OF CARBONATE OF LITHIA. (Pharmaceutical Jour. Sept. 1844.)

This salt has been recommended by Mr. Ure, as a solvent for stone in the bladder. Whether it will really prove so we may be allowed to doubt. The following is the mode of preparing it.

"Lithia, from which the carbonate is made, was discovered by M. Arfwedson, a Swedish chemist, in the *petalite* found at Uton, in Sweden; it also exists in the *triphane* found in the same locality. To prepare the salt, the mineral containing the lithia is first to be porphyrized, and then calcined in a platinum crucible, with five times its weight of pure nitrate of barytes. The product of the calcination is to be immersed in from fifteen to twenty times its weight of water, and hydrochloric acid added in excess, which entirely dissolves it. Evaporate the solution to dryness; treat the residue with water acidulated with hydrochloric acid, and filter to separate the silica. Into the filtered solution pour sulphuric acid in excess, to throw down the barytes; saturate the liquor, from which the sulphate of barytes has been separated, with ammonia, which throws down the alumina; and the liquor containing sulphate of lithia with some ammoniacal salts, is to be evaporated to dryness, and calcined so as to drive off the ammoniacal salts.

"The residue, which consists of sulphate of lithia, is to be treated with water, the solution filtered, concentrated, and precipitated with carbonate of soda. The precipitate should be washed with a very small quantity of water.

"Carbonate of lithia is found in solution in some mineral waters, and more especially in those of Bohemia; it is also found in some of the mineral waters in France. We can readily understand, therefore, the cause of the advantage derived from these waters in the treatment of patients affected with gravel, as well as those troubled with calculi of the bladder."

---

### PRINCE LOUIS BUONAPARTE'S VALERIANATES.

If the Buonaparte family had never been employed in a worse manner than Prince Louis is now, it would have been better for mankind, though perhaps worse for them. Certainly they would never have been *Princes*, had they confined themselves to making preparations of quinine and zinc. The greatest of the name was chiefly given to *steel.*

Prince Louis, however, has communicated, in the Journal de Chimie Médicale, his methods of preparing the valerianates of quinine and zinc. Nothing is said of the medicinal properties of the salts. Those who are curious pharmaceutically, we must refer to the number of the Pharmaceutical Journal for September.

---

### DR. HOULTON ON DIGITALIS.

Dr. Houlton read a paper on Digitalis Purpurea, before the Medico-Botanical Society, which has been reported in the Pharmaceutical Journal for September, and contains some valuable suggestions. These we shall extract.

"The parts of the plant to be employed in medicine named by authors, are the root, flowers, and seeds. Should any one wish to employ the root, the rule Dr. Houlton has given on various other occasions should be borne in mind, namely, the roots of biennial plants are to be collected in the autumn or winter of the first year of their duration; this rule he has never seen in any book, but it is an absolute one. Dr. Withering mentions, that Dr. Crowley, of Cambridge, was cured empirically of hydrothorax by the root, after regular practice had failed. The leaves of this plant, like the leaves of all biennials, should be gathered in the second year of their duration, and as soon as possible after the first flowers have expanded. The plant with a purplish stem should be selected; the leaves, after removing the midrib, should be carefully dried, without the aid of artificial heat; no rule is needed for the collecting of the flowers and seeds. In a work of distinguished rank it is asserted, that as digitalis is a biennial plant, its fresh leaves may be procured at any season of the year. Now this is not from its being a biennial merely, but from the circumstance of its being a very hardy biennial. The leaves of biennial plants die in the winter; in mild winters and in sheltered situations the leaves of digitalis will remain through the winter.

"Leaves have been offered for sale as digitalis, which were those of verbascum nigrum; they have much of the general appearance of digitalis leaves, and might deceive the unwary; other species of verbascum have leaves somewhat similar to those of digitalis.

"The usual preparations of the leaf are the powder, tincture, infusion, fluid, juice and extract. Dr. Houlton thinks no others are needed than the powder and the tincture, for the more simple we keep our vegetable Pharmaceutical preparations the better. It is worth noticing the changes that have taken place in the preparations of the tincture and infusion in the present Pharmacopœia; they are both weaker than in the former Pharmacopœias. The relative strength of the two tinctures is as 8 is to 10, and that of the infusion is as 8½ is to 21—here is a great difference; it is important to bear this in mind, when we compare the practice of authors who wrote previously to 1836 with those who have written since. The infusion of the present Edinburgh Pharmacopœia is double the strength of that of the London. The opinions of authors as to the dose of this medicine are curiously different. The ordinary dose of the tincture, according to those who are accounted the best authorities, is from ten to forty minims. Dr. Pereira states, he has frequently given a drachm of the tincture of the best quality three times a day for a fortnight, without observing any marked effect. He adds, 'I know that some practitioners employ it in much larger doses, as an ounce or half an ounce, with much less effect than might be imagined.' Dr. Pereira also states, that Mr. King, of Saxmundham, in Suffolk, gives from half an ounce to an ounce of the tincture in some cases with decided advantage; and that he, Mr. King, has given as much as two drachms to a child nine months old. Mr. King lives in Suffolk, a county in which we are informed the plant does not grow. He would like to know something of the history of Mr. King's tincture. Dr. Houlton would not advise any one to try such doses, prepared after the plan proposed in this paper, that is, from the genuine purple-stalked digitalis. He has seen very unpleasant effects from ordinary doses, when continued several days, and is inclined to believe that the discrepancies amongst authors in reference to the dose of this very important remedy, arise from the want of uniformity in the preparations they employ; hence they write on different things under the same names, and discordant statements are the necessary consequence."

---

### Destructive Consequences of the Indiscriminate Sale of Poisons.

In the last number of the Pharmaceutical Journal there are two letters on this subject, which deserve attention. One druggist writes that in the *Times* of that morning are four cases of poisoning by arsenic, three of them fatal. He protests: "that he never sells arsenic, nor substitutes any other preparation when arsenic may be asked for, as I consider this latter practice highly objectionable. Even when asked for 'fly-powder poison,' I have a great objection to supply this preparation, and frequently refuse it to the party who may ask for it, unless very well known to me. There are many other preparations which I think ought to be also denied, such as laudanum, prussic acid, sugar of lead, crow-fig, syrup of poppies, oxalic acid, and many others." This is, perhaps, going rather too far. Another Druggist, adverting to a case of poisoning by oil of bitter almonds, dwells on the careless manner in which chemists and druggists sell this article in small quantities, in a state of much greater concentration, for culinary purposes, than is safe.

He observes: "The use to which it is applied for flavouring spirit, has not only opened the door to its sale among large consumers, but any body may now a days obtain a little who has a mind to make his own ratafia or noyeau. The more objectionable form however to me is, the one from which the nearly fatal case alluded to above resulted, and that is the sale of the essence (as it is called) of almonds, where a few drops are directed as sufficient to flavour a pudding or custard. Now when we consider the hands into which these said essences generally come, and the careless way in which cooks are accustomed to guess at their proportions, and how little used they are to the word 'Guttatim'—I do think it would be much safer to supply the public with a more diluted article—in which spoonfuls might be used, and when the tempting flavour of the bottle, left carelessly about, would not involve anything like so much danger to those who like to have a taste of what smells so very nice."

He has known persons enter a shop for a pennyworth of morphia, colchicum being as much in demand as laudanum. This Druggist too, is in favour of the sale of poisonous substances being much more restricted than it is. All poisonous articles, for whatever purpose bought, should have "poison" labelled on the bottle, &c. Unfortunately some cannot read, others will not, and still more do not pay attention. The druggist is, at least, bound to caution his customers on the nature of what they are purchasing. The safest plan, no doubt, would be to refuse to sell poisons, in whatever quantity, without a knowledge of the parties, and something like a guarantee of their purposes. In large quantities poisons should only be vended with the sanction of a medical certificate.

---

ON THE TREATMENT OF CERTAIN AFFECTIONS OF THE NECK OF THE UTERUS BY THE ACTUAL CAUTERY. By Dr. E. LABORÉE. (L'Expérience, Août 22.)

This Essay consists principally of an exposition of the views and practice of M. Jobert. It appears that M. Jobert was encouraged first to practise this operation by the fact that the numerous nerves distributed to the uterus, on arriving at that part of the neck to which the vagina is attached, abandon the uterus, and are distributed to the walls of the vagina, so that the vaginal portion of the neck of the uterus is found to be destitute of nerves, as if the insertion of the vagina formed an impassable barrier.

1. *Cases in which the Actual Cautery may be had recourse to.*—With the exception of those slight ulcerations which accompany certain acute or chronic discharges, and which cease as soon as the principal affection is vanquished, and with the exception also of ulcerations of a syphilitic nature, almost all ulcerations affecting the neck of the uterus are susceptible of being attacked by the actual cautery. Thus, M. Camille Laurès enumerates amongst those cases which require cauterization, ulcerations which are exuberant or fungoid, or deep; which are complicated with bleeding, with simple hypertrophy, or with engorgement with softening or induration of the organ. Nay more, occasionally M. Jobert applies the caustic where no ulceration exists.

It not unfrequently happens that patients present an excess of volume of the neck of the uterus giving rise to numerous inconveniences, such as a state of habitual irritation of the vaginal mucous membrane—a painful pressure permanently exercised upon the rectum, for most frequently the uterus is in a state of antero-version—pain in walking—lassitude—lumbar pains, &c.

These symptoms, which frequently resist ordinary treatment, are, according to the author, promptly relieved by the application of the cautery to the neck of the uterus, and this is explained by the fact, that all the inconveniences experienced by the patient, are attributable to the excess of volume of the organ.

Another affection of the neck in which cauterization acts altogether as a specific, consists in the formation upon the surface of the organ, most frequently inside the lips, of a veritable erectile tissue, bleeding with the greatest facility on the least touch or the slightest irritation. In cases of this kind the patients soon acquire a chlorotic tint, which by degrees gives to the physiognomy that peculiar aspect so generally indicative of cancerous affections; a few cauterizations destroy the new tissue, and the constitution speedily recovers itself as soon as the exhausting cause is removed.

Finally, cancer, with or without uleration, when it is limited to the neck of the uterus, ought especially to be treated by the actual cautery, and we, (*the author of the essay*) should not hesitate to give in every case the preference to this mode of destruction.

*On the Mode of employing the Cautery.*—For this purpose you employ iron rods terminating in an expanded extremity, and a speculum of ivory to protect the surrounding parts, the ivory being a bad conductor of caloric. After having wiped off with a piece of lint the muco-pus which covers the ulcerated surface, you apply the iron, taking care that it is at a white heat, in order that it may not adhere to the parts with which it comes in contact, or, in withdrawing it, you may pull the eschar which it has made.

*On the Mode of Action of the Cautery.*—The first effect is, and ought to be, the production of an eschar. Care ought to be taken that the iron be at a white heat, as otherwise the disorganization is not so complete, and more irritation is produced. The colour of the eschar is greyish; its extent may be, without fear, as considerable as that of the surface of the neck of the uterus.

Generally speaking, the patient does not experience, either immediately or consecutively, the slightest re-action. The whole power of the remedy becomes concentrated and exhausted on the place on which it is applied. The patient does not feel the cauterization at the time; she will not experience any ill consequence from it afterwards. Though the patients, however, experience no sensation of burning, they do occasionally suffer real pain—this pain resulting (according to the author) from the pressure made through the medium of the insensible or vaginal portion, upon the sensible or supra-vaginal portion of the neck of the uterus, which in these cases is diseased also.

Pain is experienced from the actual operation only in two cases; viz. 1st. If, by accident, a part of the vaginal parietes has been left uncovered, and which is then burnt by radiation; 2ndly, if it should be necessary to introduce the iron so deeply into the orifice of the neck as to arrive at the level of the supra-vaginal part. After the operation, the patients return to their beds as if they had only been undergoing a simple examination by the speculum. Out-patients even proceed at once to their homes. The re-action is so completely limited to the part touched, that menstruation is in no degree impeded by the operation. Several women cauterized on the evening preceding their usual monthly period, have seen the discharge appear as usual.

In eight or ten days time, the operation can be repeated, as most usually the slough has then separated. In its place is seen a lovely roseate suppurating surface, and the neck is diminished in volume. Sometimes the eschar is more slow in separating, but this is about the usual period.

After the required effect has been obstained from the operation in cases of hypertrophy of the neck, cicatrization is allowed to take place. There is nothing particular to say on the manner in which this takes place, it is completed generally in about twelve or fifteen days after the last cauterization, when the wound becomes covered with a delicate and transparent pellicule. When the operation has been performed for the purpose of destroying ulcerations, the application of the cautery ought not to be given up until the wound presents an even surface, without projections, and its aspect is reddish, uniform, and the liquid which it secretes is simply purulent.

MM. Laborie and Laurès both assert that this operation may be had recourse to without fear, as in no case has the slightest painful re-action followed the cauterization. Is it an infallible remedy? Undoubtedly not; but never, say they, can you find a more powerful remedy, and never will bistouri be able to destroy more effectively or more surely that which a healthy surgery ought not to allow to continue.

---

### Cheiloplastic Operation. By Thomas D. Mutter, M.D.
(Medical Examiner, Philadelphia.)

The lower lip, from its conspicuousness, its utility in articulation, and also in the prevention of an involuntary and incessant flow of saliva, forms a very important portion of the face. Unfortunately, it is exceedingly prone to diseases of various kinds, especially tumors and ulcers, requiring for their relief the removal of the whole, or a portion of the organ involved. It would be worse than useless to enter into a description of all the operations that have been devised to remedy its loss, but a brief sketch of the most novel and important may prove useful and interesting to those not familiar with this department of plastic surgery.

*Chopart's Operation.*—This operation consists in making, on each side of the diseased tissue, a perpendicular incision, which extended from the margin of the lip to a point below the base of the lower jaw. Dissecting up the flap inclosed between the incisions, he carefully removed from its upper margin all the affected tissue, either by a *transverse* or *curvilinear* cut. Then, pulling upon what remained of the flap, he brought its upper edge to the level of the margin of the natural lip, and there retained it by suture, straps, and placing the head of the patient in such a manner as to prevent all strain upon the part. This method, Dr. Mütter remarks, though apparently simple and easy of execution, does not generally answer, in consequence of the subsequent contraction of the tissue.

*Horn or Roonhuysen's Operation.*—If the part to be removed be small, the common V shaped incision is sufficient, and the parts may be brought together as in the operation for hare-lip; but where the mass is large, this process is sure to diminish the orifice of the mouth, and thus give rise to deformity and inconvenience. To obviate all this difficulty, it was proposed by Horn to detach the adjacent parts by free dissection from the maxillary bones, which would of course afford more material for the lip. The objection to this method is, that in many cases the orifice of the mouth is rendered so small as to be almost useless, besides presenting great deformity.

*Operation of Dupuytren.*—This, in ordinary cases, was nothing more than cutting away by a semi-elliptical incision all the diseased tissue. Granulations spring up from the margin of the healthy skin, occupy in part the place of the original lip, and conceal to a certain extent the deformity. It is only in mild cases, however, that such a measure could succeed.

*Celsian Operation.*—Having carefully removed the diseased part by a V shaped incision, proposed to divide the tissue remaining *horizontally*, carrying the cuts as far into the cheek on each side as be deemed necessary, after the manner of Horn; but, in order to take off the strain from the flaps, he made a *semilunar incision* in the cheek, just beyond the base of each. This enabled him to bring the parts together without difficulty; and the only objection to this operation is the danger of wounding the larger vessels, nerves, and ducts of the cheek, in making the semilunar incisions.

*Operation of M. Serres.*—If the disease, as is sometimes the case, is confined to the integuments and subjacent muscles, leaving the mucous lining of the lip sound, Serres cuts away only the affected part, and then turns the mucous membrane over the margin of what is subsequently to form the lip. A few stitches are sufficient to hold it in place, and as union by the first intention usually occurs, a very natural and useful organ may thus be made. This method, however, will only answer in cases of superficial and recent disease.

*Operation of T. W. Roux.*—After removing the affected tissues, and forming suitable flaps of the adjacent parts, M. Roux takes away with the saw or cutting instruments the prominent centre of the maxillary bones, so as to make room for the proper and easy adjustment of the integuments intended to replace the organ destroyed. This operation is barbarous, because unnecessarily severe.

*Operation of P. Roux.*—Professor Roux, determined to surpass his namesake, saws out an inch or more of the bone, and then, by drawing the lateral flaps towards each other, diminishes the breadth of that part of the face involved in the disease. Then detaching the flaps, he draws them across the opening in the bone, and the sutures which hold the soft parts are generally sufficient to hold the bones in their proper places.

*Operation of Mr. Morgan.*—This consists in *first* removing the entire lip by a semilunar incision, the concavity of which is uppermost; and *second*, in making an incision also curvilinear and parallel to, and about an inch or more below the first. The skin included between the two is then carefully detached, except at its extremities, and lifted into the place occupied by the diseased lip.

*Operation of M. Blasius.*—M. Blasius has performed a very simple operation, when the tumor was large: and, according to his statement, with decided success. After removing the diseased mass by a common V shaped incision, he next divided the integuments along the base of the lower jaw by two incisions, which commenced at the entering angle of the V, and extended an inch or more in the direction specified. Lifting the flaps, he made them occupy the place of the original lips.

*Operation of Dieffenbach.*—This surgeon has recommended an operation apparently hazardous and severe.

"Having pared away the useless remains of the former diseased lip, or separated the cicatrized margin, a horizontal incision, about two inches long, is carried from either angle of the mouth outwards, through the cheeks, so as to throw the mouth widely open. The length of these incisions must be regulated according to the width of the mouth; or, as a general rule, the combined incisions must somewhat exceed in length the breadth of the upper lip. From the outer point of each of these, another incision is next carried obliquely downwards and towards the median line; the section in this case likewise extending through the whole thickness of the cheek. Thus, by means of the first operation for paring the cicatrix, and by the succeeding horizontal and vertical incisions, a flap will be prepared on either side to replace the defective lip; this flap is of a quadrangular form, and maintains a connexion of more than one inch wide with the soft parts covering the tissue of the lower jaw. It may be useful further to separate the mucous membrane at its attachment to the gums to allow of the more ready traction of the flaps."

The severe injury inflicted on the facial nerve, the large arteries and veins, and possibly the parotid duct, has rendered this operation anything but popular.

*Operation of Liston.*—This consists in first removing the diseased mass by a horizontal and two perpendicular cuts, or by one curvilinear in shape; and, second, in detaching a flap from the chin and neck, twisting it on its pedicle, placing it in the seat of the original lip, and there retaining it by suture. After adhesion has taken place, the pedicle is divided, and a wedge-shaped piece removed so as to allow the flap to be laid down smoothly. This method, it is obvious, is frequently applied to the restoration of other parts, and will answer here exceedingly well in many cases. Dr. Mütter, however, prefers the following operation, "as there is *less scar*, and less risk of sloughing of the flaps."

*Dr. Mütter's Operation.*—Having first removed the diseased mass by a semi-elliptical incision, two slightly curved incisions are carried from the centre of this line, downwards and outwards, to the base of the inferior maxillary bone. Then, from the terminal extremities of these incisions, two others are carried upwards and outwards along the base of the lower jaw until they reach a point opposite the initial and terminal points of the original semilunar incision. Two quadrangular flaps are thus marked out, and immediately detached from the subjacent bone. These flaps are then raised and placed in the position originally occupied by the lower lip, and then united to each other at the mesial line, and also by their lower thirds to the triangular piece of integument, (left between the two lines which started from the centre of the semi-elliptical incision) by means of the twisted suture. By the elevation of these flaps, a raw surface is left on each side to heal by the modelling process or by granulation.

---

ON THE TREATMENT OF THE INFLAMMATORY AFFECTIONS OF MALARIOUS DISTRICTS. By WILLIAM M. BOLING, M.D. (American Journal of Medical Sciences.)

Dr. Boling considers that a peculiarity of the febrile excitement produced in the system by, and accompanying local inflammations in those residing in marshy districts is, that it has a tendency to assume the intermittent or remittent type, as malarious fevers not connected with local inflammations have. Another striking peculiarity about these inflammations, he continues, is the obstinacy with which they resist what is *generally considered* purely antiphlogistic treatment.

The treatment adopted by Dr. Boling consists in the administration of free doses of quinine, not indeed as the sole remedy, but combined with the use of gentle laxatives occasionally, and mild antiphlogistics. Under these remedies it appears that fewer fatal cases occurred, and convalescence was rendered less tedious, than under the usual line of treatment; at which we are the less surprised, as it appears that, according to the old system, the physicians used to "take pride in boasting of their hundred-grain doses of calomel, or the number of *drastic* pills given in a dose."

The Dr. candidly confesses that he has been able to form no very satisfactory opinion as to the modus operandi of quinine; "the observations of one day generally altering or modifying the opinions predicated upon the experience of a previous day." As a general rule, however, he considers it to act as a sedative or contra-stimulant; "more certainly reducing and controlling the action of the heart and arteries than any remedy with which I am acquainted." In some cases, on the other hand, it appeared to act as a stimulant.

A great many cases are related in which quinine was administered, usually in doses of about forty-eight grains in the twenty-four hours. As in one of these cases in which the quinine was administered, apparently with benefit, it was pretty evident that malaria had no agency, the author inclines, towards the end of the Essay, to alter the opinion with which he set out, viz., that it was only on patients labouring under the poisonous influence of malaria that the sedative action of the medicine was exerted, and to consider "that under any circumstances the quinine may be made to act as a sedative by merely proportioning the dose to the degree of inflammatory action going on in the system at the time."

The Dr., not wishing to ride his hobby very severely, concludes by confessing that, "as an antiphlogistic remedy in elevated and healthy localities, it (quinine) will probably never supersede the lancet, antimonials, &c., though it may, in many cases, be brought to their aid; but in malarious regions, ere long, it will generally be looked upon as the safest and most manageable contra-stimulant we possess, and at the same time one sufficiently powerful, while other agents of the same class will only be used to fulfil some casual indication, or as adjuvants to this, the principal remedy."

---

ON AN ACCIDENTAL FIBROUS TISSUE OF THE NECK OF THE UTERUS, AND PARIETES OF THE VAGINA. By Dr. E. PERAIRE, of Bourdeaux. (Gazette Médicale de Paris, Aôut 31).

The distinctive character of this formation, according to the author, is, that whatever the part on which it forms, the accidental fibrous tissue presents a membranous aspect. It may assume various and very irregular forms; sometimes there are found concentric layers which envelope the neck of the uterus, and assume a capsular appearance. At other times, the membrane takes an angular form; there then appears, starting from a point perfectly distinct from the neck of the uterus, a fold which, traversing its tissue, is extended upon a portion of its surface, and forms, by the separation of the fibrillæ which compose it, an angle of varying degree; the disposition of this tissue may be compared with that which is seen in the ocular conjunctiva at the inner canthus, when it takes an angular form. Finally, the accidental fibrous tissue presents itself in the vagina in the form of separate or united bands.

With regard to the manner in which these tissues are formed, the author does not appear to be at all clear; he thinks, however, that in several cases of inflammation of the body or neck of the uterus, or of the parietes of the vagina, he has found a state of engorgement of the sub-cutaneous glands, and that this engorgement was of such a nature as to provoke a secretion somewhat resembling varnish, extending itself upon, and even beyond, the diseased parts.

If this fibrous tissue occupies a considerable surface, and has taken the capsular form, it may cause atrophy of the neck of the uterus. In other cases, where it is of less extent, it occasions changes of position in the uterus, either total or partial; it may also give rise to a modification of nutrition in the affected organ.

*Treatment.*—As this accidental tissue has a great tendency to contract, and become an inelastic band which impedes the free exercise of certain functions, the author considers that prompt treatment is requisite. The administration of medicines internally is of but little service; the author recommends therefore an early recourse to surgical operation. If the tissue present the capsular aspect, the membrane must be circumscribed by a simple circular incision, along its greater circumference, taking care that the incision along the neck of the uterus, and not at its implantation into the vagina, and still less on the vagina itself; the incision ought not to be of any depth. After the blood has been allowed to flow for a short time, the wound is to be touched with nitrate of silver; and some simple dressing applied. Suppuration soon becomes established, and the membrane is detached either entire, or in fragments after a short time. This operation is more complicated than when it is only necessary to incise the tissue at one point of the circumference of the neck or of the vagina. For partial adhesions of the uterus to the vagina, a straight bistouri is used to cut from without inwards, and a curved bistouri in other cases. It is necessary in all cases to respect the recto-vaginal septum behind, the vesico-vaginal septum in front. If any membranous bridles exist in the vagina they must be destroyed either by the long scissors or by the bistouri.

